# Conserved regulatory motifs in the juxtamembrane domain and kinase N-lobe revealed through deep mutational scanning of the MET receptor tyrosine kinase domain

**DOI:** 10.1101/2023.08.03.551866

**Published:** 2023-08-03

**Authors:** Gabriella O. Estevam, Edmond M. Linossi, Christian B. Macdonald, Carla A. Espinoza, Jennifer M. Michaud, Willow Coyote-Maestas, Eric A. Collisson, Natalia Jura, James S. Fraser

**Affiliations:** 1 Department of Bioengineering and Therapeutic Sciences, University of California, San Francisco, San Francisco, United States; 2 Tetrad Graduate Program, University of California San Francisco, San Francisco, United States; 3 Cardiovascular Research Institute, University of California San Francisco, San Francisco, United States; 4 Department of Cellular and Molecular Pharmacology, University of California San Francisco, United States; 5 Helen Diller Family Comprehensive Cancer Center, University of California, San Francisco, United States; 6 Department of Medicine/Hematology and Oncology, University of California, San Francisco, United States; 7 Quantitative Biosciences Institute, University of California, San Francisco, United States, United States

## Abstract

MET is a receptor tyrosine kinase (RTK) responsible for initiating signaling pathways involved in development and wound repair. MET activation relies on ligand binding to the extracellular receptor, which prompts dimerization, intracellular phosphorylation, and recruitment of associated signaling proteins. Mutations, which are predominantly observed clinically in the intracellular juxtamembrane and kinase domains, can disrupt typical MET regulatory mechanisms. Understanding how juxtamembrane variants, such as exon 14 skipping (METΔEx14), and rare kinase domain mutations can increase signaling, often leading to cancer, remains a challenge. Here, we perform a parallel deep mutational scan (DMS) of MET intracellular kinase domain in two fusion protein backgrounds: wild type and METΔEx14. Our comparative approach has revealed a critical hydrophobic interaction between a juxtamembrane segment and the kinase αC helix, pointing to differences in regulatory mechanisms between MET and other RTKs. Additionally, we have uncovered a β5 motif that acts as a structural pivot for kinase domain activation in MET and other TAM family of kinases. We also describe a number of previously unknown activating mutations, aiding the effort to annotate driver, passenger, and drug resistance mutations in the MET kinase domain.

## Introduction

**Figure 1.**
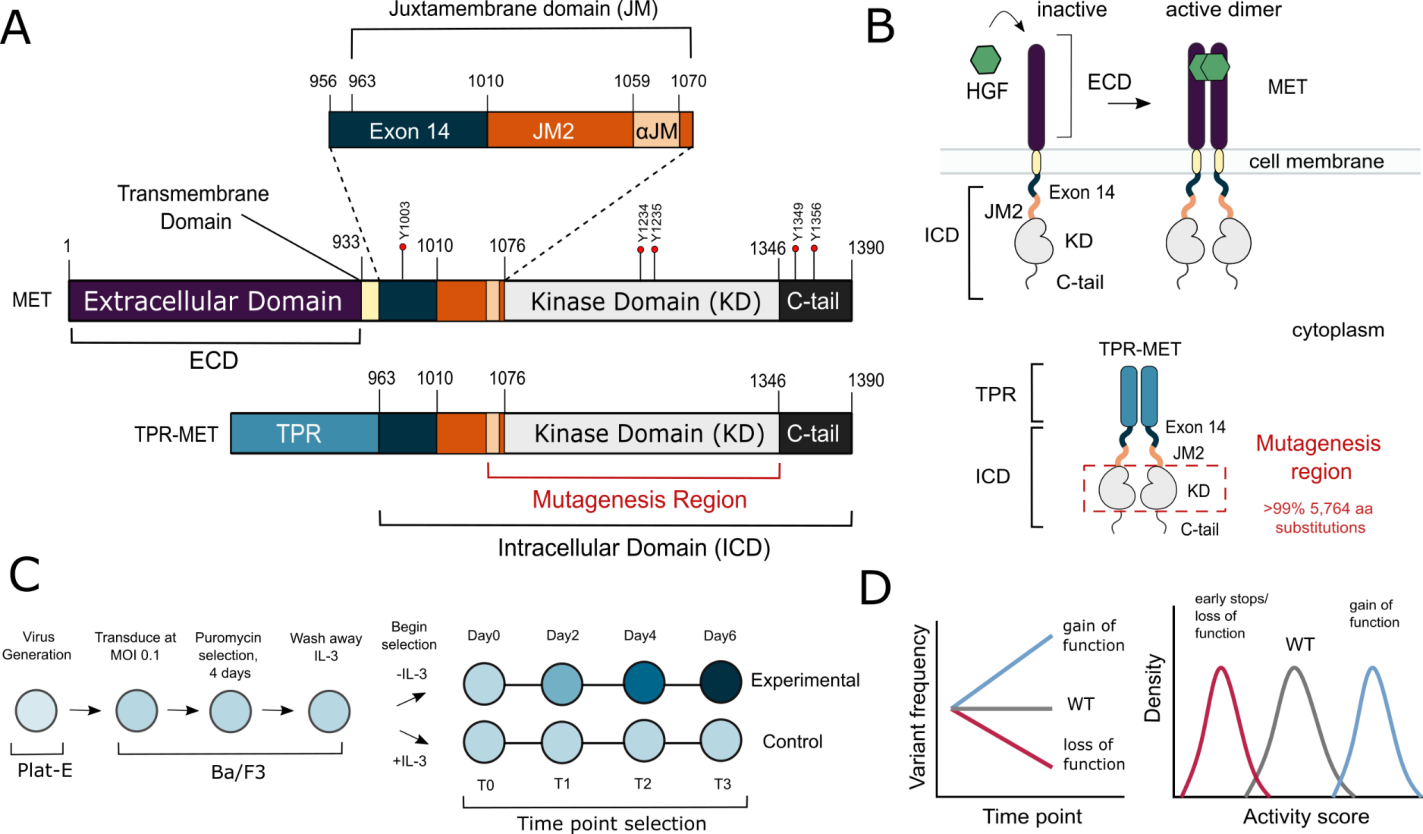
MET domain boundaries and DMS experimental workflow. (A) Domain boundaries of the full-length MET receptor (MET) and TPR-MET fusion (TPR-MET). Extracellular domain (ECD) and intracellular domain (ICD) are distinguished with important phosphorylation sites highlighted in red. Juxtamembrane domain (JM) boundaries are sectioned to annotate the exon 14 coding region and the remainder of the JM (JM2), which includes an αJM-helix (αJM). (B) Schematic of the full-length, membrane-associated MET receptor with posited MET ECD dimerization upon hepatocyte growth factor (HGF) binding; schematic of the cytoplasmically expressed, constitutively dimerized TPR-MET construct. The DMS mutagenesis region of the kinase domain (KD) is boarded in red. (C) Experimental screen workflow applied to generate and express kinase domain variants prior to selection, beginning with virus generation in Plat-E cells, transduction into Ba/F3 cells at a 0.1 multiplicity of infection (MOI), puromycin selection to enrich for positively infected cells, followed by the IL-3 selection process and time point collection for deep sequencing. (D) Post-selection method for analyzing and validating activity scores based on observed variant frequencies at each time point, measured as a slope which can then be plotted as a distribution.

Receptor tyrosine kinases (RTKs) are transmembrane proteins that play an essential role in the initiation and regulation of signaling pathways (Lemmon and Schlessinger., 2010). Most RTKs are activated upon extracellular ligand binding, promoting a relay of intracellular phosphorylation events that drive signaling (Lemmon and Schlessinger., 2010). Mutations that allow RTKs to signal independent of ligand or other typical regulatory mechanisms are commonly identified in cancer (Duplaquet et al., 2018; Saraon et al., 2021; Comoglio et al., 2018). The transition from physiological ligand-dependent to pathological ligand-independent signaling is exemplified by the RTK, MET (Figure 1A). Kinase activity of MET is normally activated by dimerization due to binding of hepatocyte growth factor (HGF) to the MET extracellular binding domain (Linossi et al., 2021). The resultant signaling is crucial for pathways implicated in development and wound repair (Trusolino et al., 2010; Petrini, 2015; Kato, 2017).

In contrast to physiological HGF-regulated activity, MET oncogenic activity arises through a variety of mechanisms such as gene amplification, mutations, gene fusions, and HGF autocrine loops (Duplaquet et al., 2018; Comoglio et al., 2018; Saraon et al., 2021; Kang et al., 2023). In MET fusions, replacement of the extracellular domain (ECD) and transmembrane domain by an in-frame translocation generates proteins with constitutive oligomerization of the intracellular domain (ICD), which leads to kinase domain (KD) activation (Sun et al., 2023; Liu et al., 2022). While MET fusions are rare in patients, they are important tools in studying MET activation in cellular models (Park et al., 1986; Vigna et al., 1999; Pal et al., 2017). A more common mutation in cancer is METΔEx14, a splicing variant that skips the entire exon 14 coding region, resulting in a shorter ICD missing approximately half of the juxtamembrane domain (JM) upstream of the kinase domain (KD) (Ma et al., 2003; Kong-beltran et al., 2006; Frampton et al., 2015) (Figure 1A). The METΔEx14 variant maintains the ligand-binding ECD and is oncogenic in part due to a combination of increased ligand sensitivity and reduced degradation due to the loss of a Cbl ubiquitin ligase interaction (Frampton et al., 2015; Abella et al., 2005; Pechard et al., 2001; Kong-beltran et al., 2006). Finally, as in many RTKs, distinct cancer-associated missense mutations are increasingly mapped to the MET kinase domain (Duplaquet et al., 2018; Chiara et al., 2003). Annotation of the status of these mutations as driver, passenger, or resistance mutations remains a significant challenge for the use of targeted therapies (Lu et al., 2017; Fernandes et al., 2021).

Here, we use deep mutational scanning (DMS) (Fowler and Fields, 2014) to screen a nearly comprehensive set of MET kinase domain mutations. Previous DMS studies have identified potential activating mutations and provided insight on the allosteric regulation of other kinases (Brenan et al., 2016; Ahler et al., 2019; Persky et al., 2020; Chakraborty et al., 2021; Hobbs et al., 2022). A phenotypic and inhibitor resistance DMS in the Ser/Thr kinase ERK2, reported tumor-associated mutations enriched at recruitment domains, in addition to identifying mutations that confer resistance without direct drug-protein interactions (Brenan et al., 2016). Similarly, when compared across Ser/Thr kinases like ERK2 and BRAF, and Tyr kinases like EGFR and ABL1, screens against CDK4/6 highlighted a “pocket protector” position near the ATP-binding site, and a generalizable allosteric, activating “keymaster” position within the N-lobe of kinases as sites of drug resistance (Persky et al., 2020). DMS of the Tyr kinase, SRC, elucidated a coordinated role between the αF pocket and the SH4 domain to stabilize SRC’s closed conformation (Ahler et al., 2019). Moreover, mutations to SRC autoinhibitory regions were identified as general resistance hotspots (Chakraborty et al., 2021). DMS of an ancestral reconstruction of the Syk-family kinases, AncSZ, revealed mutations that improve bacterial protein expression, in addition to finding commonalities between AncSZ and eukaryotic Syk kinases at regulatory regions like the αC-β4 loop (Hobbs et al., 2022). Together, these studies have been critical in illuminating novel features across Tyr and Ser/Thr kinases, which we now build on for the RTK family with MET.

To identify residues that have a direct effect on kinase function, we leveraged the murine Ba/F3 cell line as a selection system. The Ba/F3 cell line has been used as a model to study RTK signaling because it exhibits: (1) undetected expression of endogenous RTKs including Met, (2) addiction to exogenous interleukin-3 (IL-3) for signaling and growth, and (3) dependence on exogenous kinase expression for growth in the absence of IL-3 (Daley and Baltimore, 1988; Warmuth et al., 2007; Koga et al., 2022). IL-3 withdrawal therefore serves as a permissive signaling switch that allows for the effective readout of variants that alter kinase-driven proliferation (Melnick et., al 2006). Here, we used the TPR fusion of the MET ICD (TPR-MET) to screen for potential activating and inactivating mutations in the KD. The TPR-MET fusion provides the advantage of studying MET’s kinase domain in a cytoplasmic, constitutively oligomerized, active, and HGF-free system (Figure 1A–B) (Cooper et al., 1984; Park et al., 1986; Peschard et al., 2001; Rodrigues and Park, 1993; Vigna et al., 1999; Mak et al., 2007; Pal et al., 2017; Lu et al., 2017; Fujino et al., 2019). This system also affords enough dynamic range to identify mutations that cause increased proliferation (Melnick et., al 2006). We also assessed the impact of exon 14 loss (ΔEx14) on the MET kinase mutational landscape to better understand this oncogenic lesion. Our comprehensive interrogation of the MET kinase domain reveals novel regulatory regions and acts as a reference for rare activating mutations in both wild type and ΔEx14 backgrounds.

## Results

### Measurement of MET kinase domain variant activities in a wild type intracellular domain

**Figure 2.**
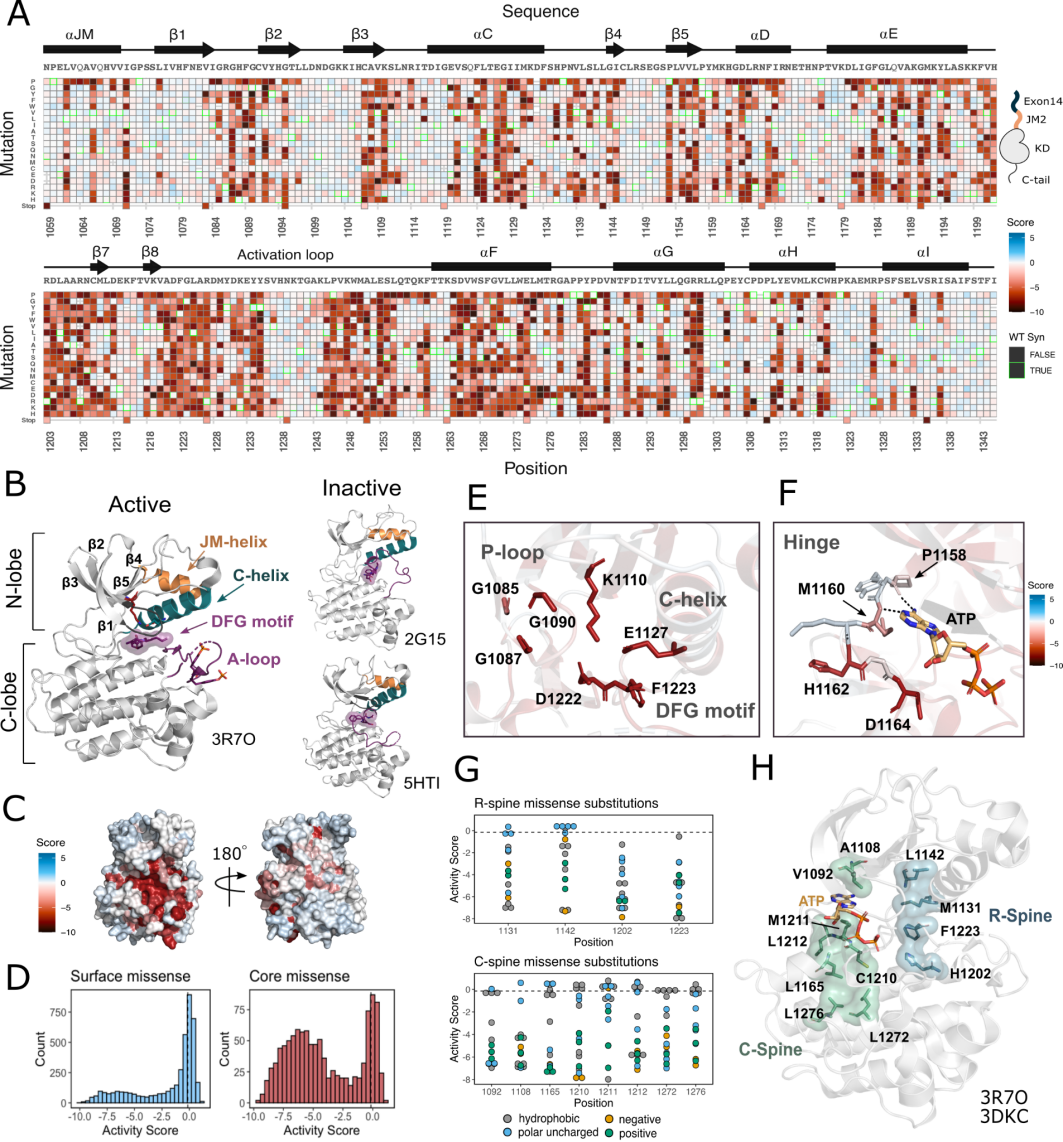
Measured effect of MET kinase domain variants across 287 amino acid positions in the context of the full-length juxtamembrane. (A) Activity score heatmap of MET kinase domain variants. WT-synonymous substitutions are outlined in green. (B) Active structure of the MET kinase domain (PDB 3R7O), and two representative inactive structures (PDB 2G15, 5HTI) with motif details highlighted. (C) Surface representation of average activity scores mapped on an active structure (PDB 3R7O). Synonymous and nonsense mutations were left out of the averaging and surface representation. Residues at the N- and C-term that were not screened, but modeled in the crystal structure are in white and not considered in the averaging and mapping. (D) Comparison of surface and core residues activity score distributions. A vertical dashed line in both graphs represents the mean score of WT-synonymous mutations. (E) Catalytic site and key residues involved in ATP binding and stabilization. Average score of variants mapped onto an active structure (PDB 3R7O). (F) Hinge region residues involved in ATP binding and stabilization. Average score of variants mapped onto an active structure (PDB 3R7O), and overlaid with the ATP molecule of the ATP-bound MET structure (PDB 3DKC). (G) Activity scores and physiochemistry of variants shown for each residue position of the R- and C- spine of MET. (H) R-spine (blue) and C-spine (green) residues highlighted on an active structure (PDB 3R7O) overlaid with the ATP molecule of the ATP-bound MET structure (PDB 3DKC).

To perform a DMS in TPR-MET, we generated a site saturation mutagenesis DNA library of the MET KD (Figure 1A). Our library also included the final alpha-helix of the JM region (αJM), which is resolved in most crystal structures, and is just upstream of the KD. Our variant library carried >99% of all possible missense mutations from positions 1059–1345 of human MET, including two internal controls: a WT-synonymous substitution at each position and premature stop codons every eleven positions (24 total). These controls allow us to use deep sequencing to estimate the fitness of WT and null variants, respectively. The library was cloned into the TPR fusion background containing the remaining JM (aa 963–1058), upstream of the αJM and KD, and the complete C-terminal tail (aa 1346–1390) downstream of the KD (Figure 1A, B). We transduced the library into Ba/F3 cells using retrovirus (Figure 1A–C). Cells were grown in the presence and absence of IL-3 and samples were deep sequenced at distinct timepoints to identify variant frequencies (Figure 1C–D). We then measured variant fitness scores using Enrich2 (Rubin et al., 2017) for each selection condition (Figure 2A).

As expected, we observed no selection in the IL-3 control condition and a low correlation was observed across replicates (Pearson’s R=0.30). In this condition, all variants, including premature stop codons, displayed near WT-like fitness (Supplemental Data Figure 1A–C). In contrast, for the condition where IL-3 was withdrawn, we observed strong evidence of functional selection. There are large differences between the fitness distributions for WT-synonymous, missense, and nonsense mutations. Nonsense mutations are strongly loss-of-function (LOF) (Supplemental Data Figure 1B,C). Very few missense mutations are more fit than the average synonymous (WT) variants, which indicates that gain-of-function (GOF) mutations are rare. In addition fitness estimates for specific mutations across replicates of this treatment are strongly correlated (Pearson’s R=0.96) (Figure 2A, Supplemental Data Figure 1B–C).

### Mutational landscape of the MET kinase domain

MET contains a canonical tyrosine kinase domain, sharing structural and conformational hallmarks with other protein kinases (Linossi et al., 2021; Schiering et al., 2003; Wang et al., 2005). Kinase domains have a conserved hydrophobic core with several motifs that are important for folding, stability, conformational transitions, and catalysis. The conformational transition between an active and inactive state relies on the hinge-like closure of the N- and C-“lobes” around an ATP-bound catalytic site. The N-lobe is a dynamic unit comprised of five β-sheets and the αC helix, while the C-lobe is a more rigid unit composed of seven α-helices connected by loops (Kornev et al, 2006; Kornev et al, 2008). Two hydrophobic “spines” (the R- and C-spine) assemble across the lobes as the kinase transitions to an active state (Taylor & Kornev, 2011; Hu et al., 2014; Taylor et al., 2015). The transitions from inactive to active states of MET are typical for a tyrosine kinase: the αC helix moves to an “in” position, positioning E1127 to form a salt-bridge with K1110. In addition, the flip of the DFG (residues 1222–1224) motif aligns F1223 with the rest of the R-spine and permits hydrogen bonds between D1222 and ATP (Kornev et al, 2006).

Our DMS results highlight that the conserved regulatory and catalytic motifs in MET are highly sensitive to mutation (Figure 2A–D). Catalytic site residues involved in either the chemical step of phosphate transfer or the conformational adaptation to ATP binding, such as K1110, E1127, D1222, and G1224, are intolerant of amino acid substitutions (Figure 2E). Residues surrounding the kinase “hinge”, which are involved in coordinating the adenosine potion of ATP (Azam et al., 2008; Dar and Shokat, 2011), are more tolerant to mutation (Figure 2F). This tolerance is especially prominent in residues that make backbone interactions with ATP. Variants in R-spine residues are strongly enriched in loss-of-function (LOF) fitness values. This result speaks to the importance of residue identity, not just physicochemical characteristics in the function of MET. R-spine residues F1223 and H1202 are part of the catalytic DFG and HRD motifs and do not tolerate any mutations (Figure 2G–H). For R-spine residues M1131 and L1142 positions, only a small number of polar uncharged substitutions show WT-like fitness (Figure 2G–H). Surprisingly, the MET C-spine was only moderately sensitive to mutations, with most hydrophobic and polar uncharged amino acid substitutions showing WT-like fitness (Figure 2G). We performed a sequence alignment of human RTK kinase domains for both the R- and C-spines, referenced to the MET kinase domain sequence (Supplemental Data Figure 2) to explore whether there was greater evolutionary conservation for the R-spine than the C-spine for MET. Consistent with our results, the C-spine was less conserved, but is enriched for hydrophobic residues (Supplemental Data Figure 2). These results indicated that key catalytic and regulatory motifs display varying sensitivity. The R-spine and catalytic residues are more highly constrained, whereas C-spine and hinge residues can tolerate a greater number of semi-conservative mutations.

On balance, most solvent exposed and loop regions were more permissive to mutations (Figure 2C–D). However, certain key regulatory elements stand out, with stronger mutational sensitivity than might be expected based on structural features alone. For example, the αC helix-β4 loop which is important in αC helix modulation and regulation in many kinases (Chen et al., 2007; Yeung et al., 2020), showed LOF effects in agreement with the previously described “ΦxHxNΦΦx” motif (Yeung et al., 2020). Similarly, the glycine residues in the “GxGxxG” motif of the P-loop, which gates ATP entry into the active site, were intolerant to substitutions. Two other relatively immutable sites included Y1234 and Y1235, in the activation loop (A-loop) of MET. The phosphorylation of these residues is required for stabilizing the A-loop in an extended conformation that enables substrate binding and efficient catalysis (Ferracini et al, 1991; Naldini et al, 1991). Collectively, this deep mutational scan confirms the importance of canonical kinase features and provides a reference point for discovering previously unexplored features of the MET kinase.

### Critical contacts between the αJM and αC helices provide insight into potential juxtamembrane regulation of MET

**Figure 3.**
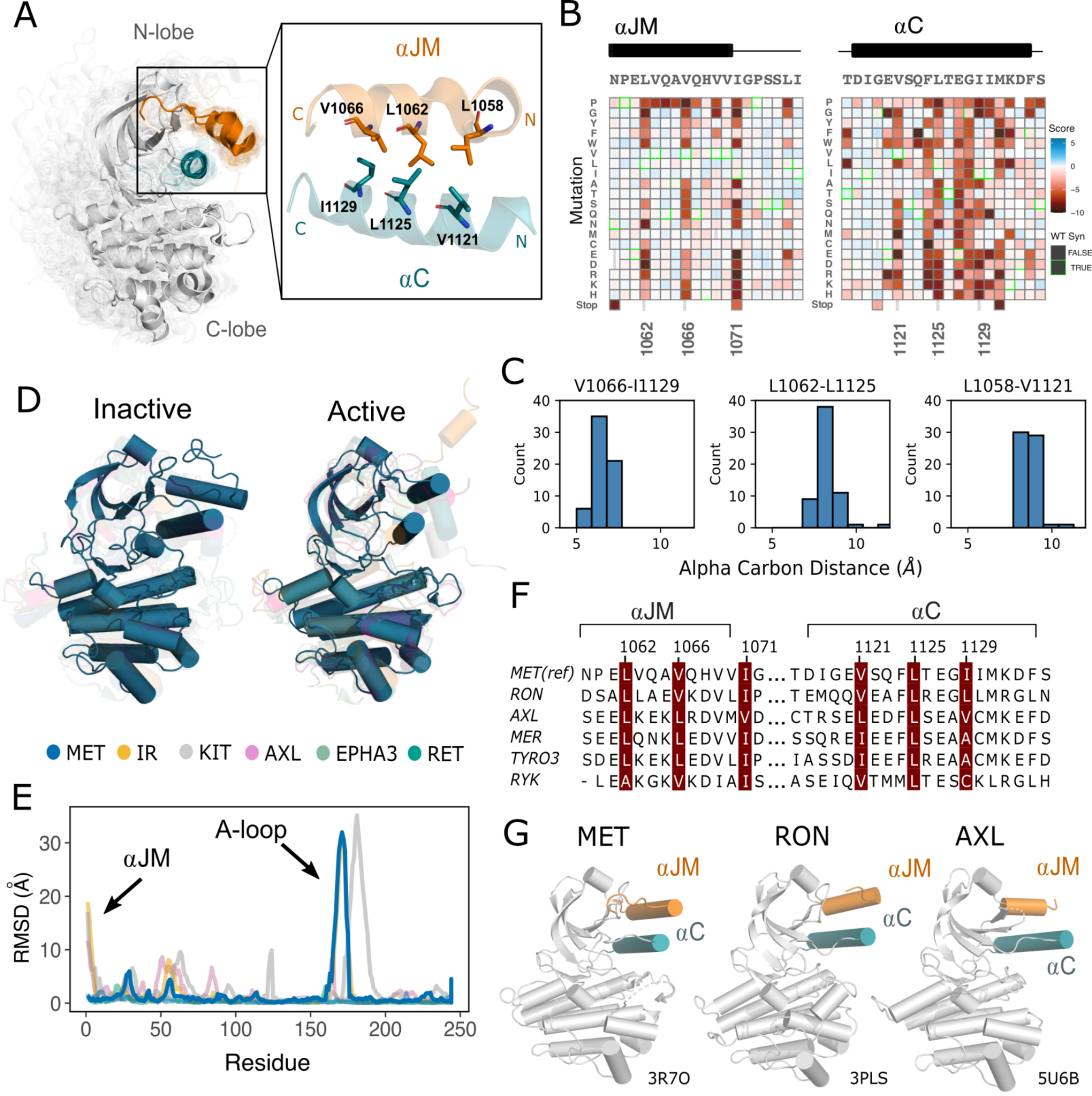
Essential αJM and αC interactions revealed through variant and structural analysis. (A) Ensemble of 93 MET kinase domain crystal structures available in the PDB. All structures, independent of conformation, were locally aligned to JM residues 1059–1070 (all resolved JM and αJM residues in orange), and αC residues 1117–1134 (teal). In solid gray is a representative active structure (PDB 3R7O). Residues involved in the αJM and αC interface. (B) Heatmap sections of the αJM and αC from the MET ICD screen. (C) Distribution of alpha carbon distances for residues in the αJM and αC interface, shown for 63 MET crystal structures in the ensemble with residues modeled for positions 1058, 1062, 1066, 1121, 1125, 1129. Distances are independent of conformation. (D) Global alignment of inactive and active RTK kinase domain structures with resolved JM regions. (E) Residue-by-residue, backbone RMSD comparisons of inactive and active structures of MET, AXL, IR, EPHA3, KIT, and RET. (F) MuscleWS alignment of human MET and TAM family juxtamembrane helix sequences. (G) Crystal structures of MET (PDB 3R7O), RON (PDB 3PLS), and AXL (PDB 5U6B) kinase domains, with αJM (orange) and αC (teal) highlighted. The inactive conformation of AXL shows an αJM and αC hydrophobic interaction similar to MET, but unlike MET, these interactions are slightly pivoted by an αJM turn in its active conformation.

Given the importance of juxtamembrane (JM) regions in controlling the activation of many RTKs (Hubbard et al 2004; Zhang et al., 2006; Jura et al., 2009; Cabail et al., 2015; Wybenga-Groot et al., 2001; Wiesner et al., 2006) and the prevalence of exon 14 skipping within the JM of MET in cancer (Lu et al., 2017), we were interested in how patterns of mutational sensitivity from the DMS could relate the JM to the activation mechanism of MET. The MET JM (aa 956–1075) is predicted to be largely unstructured, but a small region (aa 1059–1070) folds into an alpha helix (αJM) that packs on top of the αC helix of the kinase domain, forming a hydrophobic interface (Figure 3B). Since we included αJM in the DMS library, we examined the pattern of substitutions for this region and the αC helix. We observed a strong hydrophobic preference along both the αJM and αC helix residues comprising this interface (Figure 3A). The adjacent αJM residue 1071 also strongly prefers hydrophobic residues. This residue does not interact with αC helix, but evidently plays an important role in maintaining interactions with a hydrophobic patch in the N-lobe that includes residues L1076, L1097, and V1158. This result indicates the importance of burying the hydrophobic surfaces of the N-lobe, and αC helix in particular, for maintaining an active kinase in the TPR-MET background.

Since the αC helix conformation is a strong predictor of activity in kinases (Ung et al., 2018; Modi and Dunbrack, 2019), and is often modulated by protein-protein interactions or autoregulatory domains (Huse & Kuriyan, 2002), we hypothesized that these hydrophobic contacts might form preferentially in active MET kinase. The precedence for this idea comes from structural studies of RTK ICDs in the EGF, PDGF, EPH, and IR families (Zhang et al., 2006; Jura et al., 2009; Griffith et al., 2004; Wybenga-Groot et al., 2001; Wiesner et al., 2006; Li et al, 2003; Cabail et al., 2015). For example, in EGFR the JM stabilizes an active, asymmetric head-to-tail dimer (Zhang et al., 2006; Jura et al., 2009). In IR, the αJM engages with αC helix to maintain an inactive state, until αJM-αC helix interactions are released and swapped to stabilize an active kinase dimer (Cabail et al., 2015). In contrast, in FLT3 the JM packs against the catalytic cleft to stabilize an inactive KD confirmation (Griffith et al., 2004). To test how MET kinase activation is linked to JM conformation, we compared the 93 crystal structures of the MET kinase domain with a modeled portion of its JM, as a MET pseudo-ensemble (Figure 3B). We aligned these structures on the N-lobe to eliminate the effect of the changes in relative N- and C-lobe alignment that occurs with activation. Despite large changes in the relative position of the C-lobe, corresponding to active and inactive structural hallmarks, there was little αJM conformational variability or dependence on active/inactive state **(3A,C).** Rather, αJM consistently packed against αC helix and maintained the hydrophobic interface across all structures (Figure 3A–C).

To compare the MET αJM-αC helix to other RTK JM-KD interactions, we compiled a set of human RTK kinase domain crystal structures that contain a modeled αJM, and have both active and inactive structures available in the PDB. Within this set, IR and KIT displayed large conformational variability of the αJM between active and inactive states, but MET stood out again in low conformational variability independent of the kinase activation state (Figure 3D,E). Indeed, when we examined JMs and kinase domains in a protein sequence alignment of all RTK, we observed that while αC helix is conserved across RTKs, only MET (MET, RON), TAM (TYRO3, AXL, MER), and the RYK pseudokinase harbor an αJM with a hydrophobic sequence pattern (Figure 3F, G). Together, the mutational sensitivity and structural conservation of MET’s αJM-αC helix interface point to a model, that may be shared with RON and TAM family RTKs, where αJM-αC helix contacts are maintained in both the active and inactive state and are important for TPR-MET stability and activity.

### β5 P1153 is a structural pivot for the MET kinase domain N-lobe

**Figure 4.**
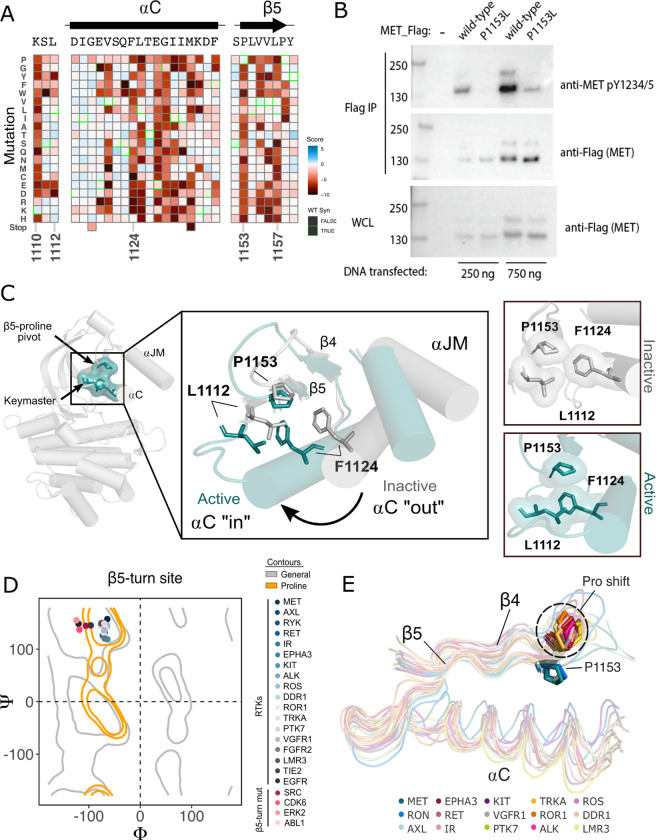
β5 Proline motif is an activation pivot for the MET kinase domain. (A) Respective sections of the MET ICD heatmap. (B) FLAG-IP western blot of the P1153L mutation post 24hr expression in HEK293 cells. (C) Residues of the MET P1153 N-lobe network displayed in an active (PDB 3R7O) and inactive structure (PDB 2G15). Surface representation of residues involved in the P1153 network. (D) Ramachandran plot and structural position of P1153 in MET and one representative kinase domain of each RTK subfamily. (E) Structural representation of the RTK Pro shift of the β4–5 loop. One representative RTK kinase domain from each sub-family is locally aligned to β4–5 of MET.

Although most regions of high sensitivity from the DMS impinge on well-described aspects of kinase activation, P1153 stood out as a previously unremarked upon position with extremely low tolerance to mutation in our DMS (Figure 4A). Only proline was tolerated in this position. P1153 is located in the β4-β5 region, which plays a role in αC helix coordination and R-spine support (McClendon et al., 2014; Meharena et al., 2013; Taylor and Kornev, 2011). The β4 strand is connected to αC helix and influences activity through regulatory mechanisms such as the “molecular break” in RTKs like FGFR, or through cis- and trans- protein interactions (Chen et al., 2007; Yeung et al., 2020). The β5 strand engages with αC helix, the hinge, and harbors the disease-associated “gatekeeper” position (Azam et al., 2008). The immutability phenotype was especially notable because P1153 is not conserved across kinases (Supplemental Data Figure 3A).

To test the importance of this residue in a more physiological membrane-bound context, we expressed WT and P11553L variants of the full-length (FL) MET receptor in HEK293 cells. Based on the DMS in Ba/F3 cells, we expected that P1153 mutations would not signal or express poorly in this context. Indeed, relative to the WT MET receptor, P1153L expressed equivalently, but dramatically reduced signals for phosphorylation of the MET activation loop tyrosines, a marker of active MET signaling (Figure 4B). To test whether this importance extended to another MET family member, RON receptor, which also has proline residue at the equivalent position, we tested and observed similar results. Notably, in RON the equivalent proline mutation also significantly compromised receptor expression (Supplemental Data Figure 3B). These experiments provided initial validation that the results from the TPR-MET construct would translate to a full-length MET receptor context, and indicated the family-specific functional importance of proline in this position ,of the β4-β5 loop.

Next we analyzed the structural environment of P1153. It packs in a hydrophobic cluster with the resistance-associated β3-αC helix “keymaster” position (L1112 in MET) (Persky et al., 2020) and F1124 of αC helix. The hydrophobic packing around P1153 by F1124 and L1112 changes across MET active and inactive structures (Figure 4C). As αC helix adopts an active “in” or inactive “out” conformation, L1112 rotates inward toward the core of the N-lobe to replace F1124 and maintain the hydrophobic environment around P1153 (Figure 4C). The maintenance of hydrophobic contacts across these conformational changes led us to re-examine the relative lack of sequence conservation of P1153 (Supplemental Data Figure 3A). We compiled a set of representative kinase domain structures from each RTK family, as well as those that have had DMS studies performed previously, and analyzed the Ramachandran angles of the analogous residue in the β4-β5 loop. Although most kinases have a non-proline amino acid at that position, the analogous residues cluster around or inside the proline-permissive contour of Ramachandran space (Figure 4D). Interestingly, while no other RTK family aside from MET and TAM family kinases have a proline at the β4-β5 loop position, most do have a proline exactly one position upstream. This shift occurs both in sequence and in structure: the upstream proline does not participate in the hydrophobic pivot observed here for P1153 in MET (Figure 4D). This analysis suggests that the MET and TAM families represent a subset of RTKs that have evolved the surrounding sequence to accommodate only proline in this structurally restrained region of the β4-β5 loop, explaining the immutability in our DMS experiment.

### Mutational landscape of the MET kinase domain in the absence of the exon 14 coding region

**Figure 5.**
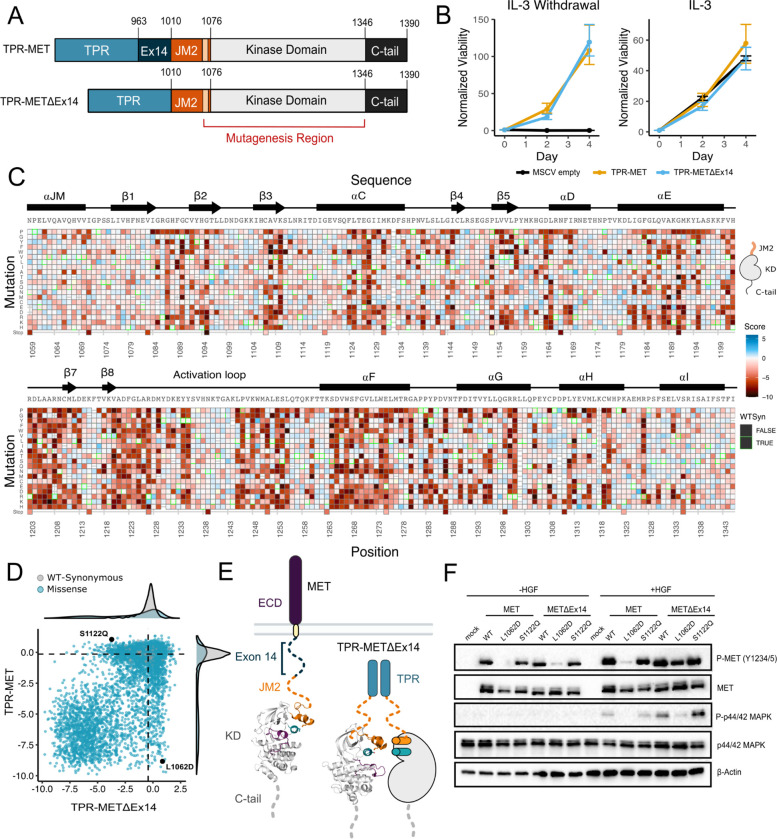
Comparative measurement of MET kinase domain variants across 287 amino acid positions in the absence (TPR-METΔEx14) and presence of exon 14 (TPR-MET). (A) Domain boundaries and schematics of the TPR-MET ICD and TPR-METΔEx14 ICD constructs. (B) Proliferation assay of parental TPR-MET, TPR-METΔEx14, and MSCV empty vectors expressed in Ba/F3 under IL-3 withdrawal and IL-3 conditions. Cell viability was normalized to day 0. (C) Activity score heatmap of METΔEx14 kinase domain variants. WT-synonymous substitutions are outlined in green. (D) Scatter plot of TPR-METΔEx14 versus TPR-MET activity scores for each variant with distributions displayed on the margins. Dashed lines represent the WT synonymous average score for METΔEx14 versus MET. (E) Schematic of the kinase domain (PDB 2G15, 3R7O) in a full-length receptor and TPR-METΔEx14 context. (F) Western blot of endogenous MET KO HeLa cells transiently transfected with L1062D and S112Q mutants in the MET and METΔEx14 receptor, with and without HGF stimulation (50ng/ml, 15 min stimulation, 37°C).

Exon 14 skipping, which maintains the ECD and transmembrane region while truncating the ICD at the JM, is one of the most common driver mutations observed in MET(Fernandes et al., 2021; Lu et al., 2017). The oncogenic effect is thought to, at least partially, result from removal of the docking site for Cbl, a ubiquitin ligase responsible for MET lysosomal degradation (Mak et al., 2007; Peschard et al., 2001; Petrelli et a., 2003; Abella et al., 2005; Kong-Beltran et al., 2006). Based on previous findings showing a TPR-METΔEx14 growth advantage over the MET receptor and METΔEx14 receptor, and TPR-MET in anchorage-independent assays of AALE cells (Lu et al., 2017) and fibroblasts (Vigna et al.,1999), we tested whether there was also a growth advantage in Ba/F3 cells (Figure 5A). Counter to expectations, we did not observe a discernible growth differential for TPR-METΔEx14 relative to TPR-MET (Figure 5B, Supplemental Data Figure 5A). It is important to note that exon 14 skipping normally occurs in the context of the full length protein, not in fusions such as TPR-MET. Therefore, the lack of a growth rate difference may be due to the need for Cbl regulation to occur at the plasma membrane (Mak et al., 2007) or due to other aspects of the Ba/F3 system. Although the lack of a growth differential means an exon 14-specific mechanism cannot be fully addressed in this experimental format, KD mutational responses are still representative and comparable.

We performed a parallel DMS of the MET KD in the background of an exon 14-skipped ICD (TPR-METΔEx14) (Figure 5C). Most regions implicated in the TPR-MET DMS were similarly sensitive in the TPR-METΔEx14 (Figure 5C,D; Supplemental Data Figure 5B). For example, the P1153 β4-β5 loop site was also intolerant to mutations in TPR-METΔEx14 (Supplemental Data Figure 5C). Similarly, the R- and C-spine were sensitive to physicochemical changes outside of hydrophobic and polar uncharged substitutions (Supplemental Data Figure 5D). To identify specific mutations of the KD that are uniquely sensitive to the presence or absence of exon 14, we filtered variants with the largest differences in activity scores between TPR-MET and TPR-METΔEx14 (Figure 5D, Supplemental Data Figure 5E).

As an example of the differences between the two backgrounds, we focused on the αJM-αC helix interface. This region displayed relatively high sensitivity to non-hydrophobic substitutions in TPR-MET (Figure 3B), but it was more tolerant to mutations in the TPR-METΔEx14 background (Figure 5C, Supplemental Data Figure 5F). To test whether this αJM and αC helix sensitivity difference translated to the full-length receptor, we introduced L1062D, a mutation at the αJM interface, and S1122Q, an αC helix surface mutation, into FL-MET and FL-METΔEx14 backgrounds in HeLa cells lacking endogenous MET (Figure 5E, F). Consistent with the results from the DMS screen, in unstimulated cells, we observed that a marker of MET activation, A-loop phosphorylation, was dramatically reduced in MET receptor for L1062D, but less so in FL-METΔEx14, relative to WT controls (Figure 5F). Furthermore, upon HGF treatment, L1062D in the FL-METΔEx14 background exhibited a high degree of activation, but L1062D FL-MET did not. An additional marker of active MET signally, phosphorylation on p44/42 MAPK (ERK), was similarly responsive (Figure 5F). In contrast, S1122Q was expected to have a gain-of-function effect in the MET receptor and a loss-of-function effect in the METΔEx14 receptor. For this mutation we observed no difference relative to WT in A-loop phosphorylation at baseline or upon HGF stimulation (Figure 5F).

These results suggest that the correlation between DMS fitness in TPR-MET and acute A-loop phosphorylation in the MET receptor is not absolute. Since KD activation at a single time point was the only parameter we explored with the membrane-associated receptor, other aspects such as sustained signaling, recruitment of specific signaling adapters, or changes in MET regulation may be more consistent with the proliferative readout of the DMS. In addition, the inconsistency in the S1122Q result may be a specific feature of the dimerised, cytoplasmic TPR-MET signaling that is sensitive to interactions with the portion of the JM deleted by exon 14. Collectively, these results highlight that the αJM-αC helix interface is sensitive to mutation and the presence or absence of exon 14 can alter this sensitivity. While the experimental parameters of this screen may limit how translatable some mutations are in a membrane-associated receptor or in non-proliferative conditions, the consistency of most residues between backgrounds provides increased confidence in the atlas of mutational effects on MET activation.

### Analysis of cancer-associated and resistance mutations in MET and METΔEx14

**Figure 6.**
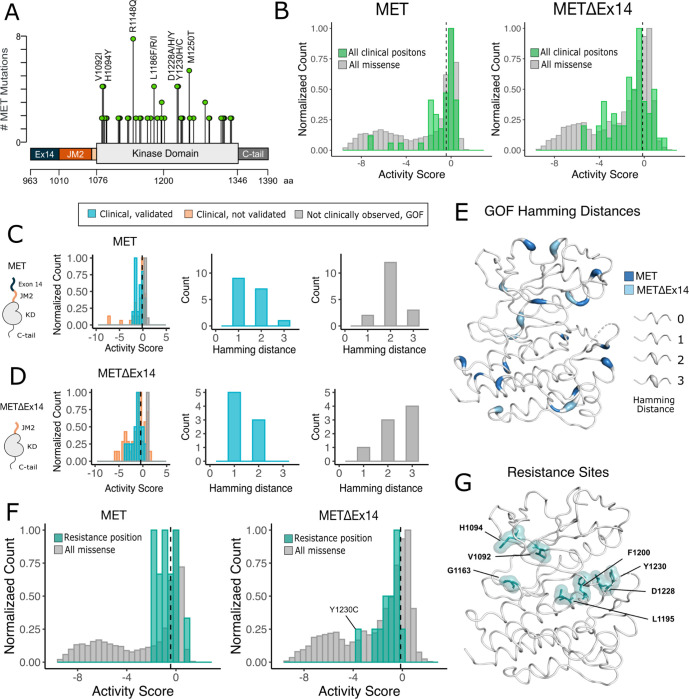
Mutations with greater proliferative effects than cancer-associated mutations, and differential sensitivities between MET and METΔEx14 identified. (A) Lollipop diagram of MET kinase domain mutations and frequencies annotated in cBioPortal. (B) Distributions of cancer-associated mutations overlaid with all missense mutations. Counts are normalized to the total mutations in each screen dataset. (C-D) Distributions of categorized cancer-associated mutations. Counts are normalized to the total mutations in each screen dataset. Hamming distance distributions of clinical, validated MET cancer mutations and clinically unobserved, GOF mutations detected in the screen for both ICD backgrounds. (E) Cancer-associated mutations mapped onto a MET kinase domain structure, colored according to MET and METΔEx14 backgrounds, with Hamming distance represented by the ribbon thickness at each position (PDB 3R7O). (F) Reported resistance mutation distributions for MET and METΔEx14, overlaid with their respective missense distributions. (G) Inhibitor resistance mutation positions shown on an active MET kinase domain structure in teal (PDB 3R7O).

To assess the ability of the DMS to classify driver, passenger, and resistance mutations, we first gathered all MET kinase domain mutations reported from clinical observations in cBioPortal (Cerami et al., 2012; Gao et al., 2013) (Figure 6A). Relative to the distribution of all missense mutations, the distribution of clinically observed mutations is strongly shifted to higher fitness values for both TPR-MET and TPR-METΔEx14 screens (Figure 6B). Most of these mutations have near WT-fitness levels, with a small number having GOF fitness effects (Figure 6B). Next we further subdivided our observations based on annotations in cBioPortal as either “clinical, validated” or ”clinical, not validated”. Notably, mutations in the “clinical, not validated” category were outliers with lower fitness values, indicating that these are likely passenger mutations (Figure 6C,D). These results validate that the DMS recapitulates known oncogenic MET kinase variants and suggest that the screen can be used to help classify driver vs. passenger mutations.

Interestingly, there are some missense mutations that are strong GOF variants in the DMS that have not been reported clinically, potentially indicative of novel activating mutations. We classify these mutations as “not clinically observed, GOF.” We hypothesized that these variants are more difficult to observe clinically because of the constraints of the genetic code. To test this idea, we calculated the Hamming distance between the WT MET codon and mutant codon for each position within our dataset (Figure 6C–E). We found that most “clinical, validated” mutations had Hamming distances of 1–2 nucleotide substitutions from WT (Figure 6C,D). However, the most common Hamming distances for “not clinically observed, GOF” codons shifted 2–3 changes away from WT (Figure 6C, D). These GOF mutations are dispersed throughout the structure of MET kinase and have distinct patterns depending on the presence or absence of exon 14 (Figure 6E). These results suggest that the DMS can identify GOF mutations that require a larger genetic “leap” than what is observed in natural populations. Furthermore, the relative paucity of “not clinically observed, GOF ” mutations at Hamming distance of 1 suggests that clinical observations have nearly identified all possible activating MET mutations that require only a single nucleotide change. Collectively, this suggests that our atlas will be of particular use for deciding on driver status for rare mutations that require multiple nucleotide changes.

Finally, we assessed the distribution of fitness effects for clinically observed resistance mutations (Duplaquet et al., 2018; Fernandes et al., 2021; Saraon et al., 2021; Lu et al., 2017; Fujino et al., 2019). Resistance mutations, which are clustered around the active site, are also enriched towards higher fitness values than the background missense mutation distribution (Figure 6F, G). This result suggests that most of these mutations can pre-exist in the population even in the absence of selective pressure from an inhibitor. GOF resistance mutations may indicate an effect on the equilibrium of kinase activation, whereas LOF resistance mutations likely affect inhibitor-protein interactions directly. For instance, Y1230C is a recurrent resistance mutation (Bardelli et al., 1998) that interrupts Pi interactions that stabilize inhibitors at the active site, but in the absence of an inhibitor is unfavorable in METΔEx14 (Figure 6F, Supplemental Data Figure 6). These results indicate that DMS has potential to interpret the effects of resistance mutations, an area of active concern for patients being treated with MET inhibitors in the clinic.

## Discussion

Our parallel DMS of the MET kinase domain has revealed how mutations affect the allosteric regulation of MET in two clinically relevant juxtamembrane backgrounds. Auto-regulation via juxtamembrane segments is a common feature of RTKs like the EGF, PDGF, EPH, and IR families (Hubbard et al 2004; Zhang et al., 2006; Jura et al., 2009). We propose that MET is similarly regulated based on the distinct sensitivity of the interface of αJM and αC helices to mutations between TPR-MET and TPR-METΔEx14. Given that exon 14 is directly upstream of this region, it is possible that the making and breaking of contacts between the αJM and αC helices during kinase activation observed in other families has a distinct structural analogue in MET. Rather than variable contacts between the JM and αC helix, as observed in other families, we hypothesize that the exon 14 region of the JM has activation-state dependent contacts with the αJM-αC unit. In this model αJM-αC helix move in unison between active and inactive conformations, with the rest of the JM making contacts to αJM that regulate the activation state. When exon 14 is skipped, these contacts are absent, tilting the equilibrium towards kinase activation. This model could augment known mechanisms of METΔEx14 activity, most notably a lack of Cbl-mediated downregulation, to enhance the oncogenic potential of this variant.

Conclusively addressing the question of how exon 14 skipping activates MET is difficult in the TPR-fusion system that we have employed here. While soluble, cytosolic oligomerization occurs for MET fusion proteins observed in some tumors, using the TPR-fusion may not accurately model all of the changes found in other types of MET lesions, such as METΔEx14. Therefore, this strategy may underestimate the effects of regulatory mechanisms related to membrane engagement or ICD oligomerization. Nonetheless, the experimental parameters of this study based on cell proliferation allowed us to understand the mutational sensitivities of the kinase domain in a cytoplasmic and constitutively active environment..

Analyzing the mutational landscape with the TPR-MET fusion approach employed here has led to multiple new insights, some of which were also validated in the full-length MET receptor. For example, one of the most unexpected regions of mutational sensitivity was P1153 in the β4-β5 loop. Based on the observed proline sequence shift, β4-β5 loop conformational restraints, and conserved hydrophobic network around the β4-β5 loop position, we suggest that this proline residue is an evolutionary dead-end. In ancestral kinases, a proline could be adopted without a significant cost at the corresponding β4-β5 loop position due to this residue being positioned in proline-permissive Ramachandran space. After drift of the surrounding hydrophobic residues, the sequence for these residues has adapted to the unique structural properties of proline, rendering it impossible to substitute for an alternative amino acid. Interestingly, evolutionarily distant kinases that do not have proline at this position exhibit inhibitor resistance mutations at this site (Brenan et al., 2016; Persky et al., 2020; Lee and Shah, 2017). In some distant kinases such as PDK1, this β4-β5 and αC helix region is part of an allosteric binding site for inhibitors (the PIF pocket) (Rettenmaier et al 2014). Given that the proline pivot region is largely intolerant of mutations, this site could potentially be targeted for allosteric inhibitors to avoid the development of resistance mutations observed in the clinic for many other small molecule MET inhibitors..

In summary, our parallel DMS of MET and METΔEx14 has built an atlas of variant effects. Moreover, we identified a small number of unique sensitivities in each background, which provides hypotheses about the mechanism of exon 14 skipping in cancer. We also observed a number of strong GOF variants that have not been observed in the clinic. Strikingly, these variants are enriched in 2 and 3 nucleotide changes, suggesting that our DMS will be especially useful in classifying rare driver mutations and that the clinical population has essentially sampled most of the single nucleotide changes. These results comprise a valuable resource for classifying driver, passenger, and resistance mutations for MET and other RTKs.

## Materials and Methods

### Mammalian cell culturing

Ba/F3 cells were purchased from DSMZ and maintained in 90% RPMI (Gibco), 10% HI-FBS (Gibco), 1% penicillin/streptomycin (Gibco), and 10ng/ml IL-3 (Fisher), and incubated at 37°C with 5% CO2. Ba/F3 cells were passaged at or below 1.0E6 cells/ml in order to avoid acquired IL-3 resistance, and regularly checked for IL-3 addiction by performing 3x PBS (Gibco) washes and outgrowth in the absence of IL-3 to confirm cell death in the parental, empty cell line.

Plat-E cells stably expressing retroviral envelope and packaging plasmids were originally gifted by Dr. Wendell Lim. Plat-E cells were maintained in 90% DMEM+HEPES (Gibco), 10% HI-FBS (Gibco), 1% penicillin/streptomycin (Gibco), 10ug/ml blasticidin, 1ug/ml puromycin, and incubated at 37°C with 5% CO2. Plat-E cells were maintained under blasticidin and puromycin antibiotic pressure unless being transfected.

HEK293 cells were maintained in DMEM (Gibco) supplemented with 10% FBS (Gibco) and 1% penicillin/streptomycin (Gibco) at 37°C in 5% CO2.

Human MET knockout HeLa cells were purchased from Abcam and maintained in 90% DMEM+HEPES (Gibco), 10% HI-FBS (Gibco), 1% penicillin/streptomycin (Gibco), and incubated at 37°C with 5% CO2

### Cloning and retroviral vectors used

Both pUC19 (Cat. 50005) and MSCV (Cat. 68469) were ordered from Addgene. To ensure unique cut sites within the vectors for introduction and shuttling of the variant library, a new multiple cloning site was introduced into each plasmid. Wild type TPR-MET-IRES-mCherry and TPR-METΔEx14-IRES-eGFP genes were cloned into pUC19 as the parental constructs for library generation and all site-directed mutagenesis. The full-length MET, METΔEx14, and RON receptor cDNAs were subcloned into pcDNA3.1 vector by Gibson assembly and incorporated a C-terminal single Flag tag sequence. All mutations were introduced by quick change mutagenesis.

### MET kinase domain variant library generation and cloning

The MET kinase domain variant library was designed to span amino acid positions 1059–1345, which contained the full kinase domain (aa 1071–1345) and a portion of the juxtamembrane (aa 1059–1070). The library was synthesized by Twist Bioscience with one mammalian high-usage codon per amino acid to prevent over-representation of specific residues. The library was received in 96-well arrays of lyophilized DNA at 50 ng per well, where each well contained all variants (missense and WT-synonymous) per position of the kinase domain. The lyophilized library was resuspended in 100uL of 1X TE buffer, and 5ng of DNA from each well was amplified with low cycle PCR to increase the starting material using the following the NEB Q5 High-Fidelity recipe per well: 10μL 5X Q5 buffer, 5ng template DNA, 2.5μL 10μM forward primer, 2.5μL 10μM reverse primer, 1μL 10mM dNTPs (2.5μM each), 0.5μL Q5 Polymerase, nuclease free water to a final volume of 50μL. The following thermocycler parameters were then applied: initial denaturation at 98°C for 30s, followed by 10x cycles of denaturation at 98°C for 10s, annealing at 62°C for 30s, extension at 72°C for 1 min, and a final extension at 72°C for 5 min. A 1% agarose, 1X TBE diagnostic gel was run with 2μL of each sample to confirm amplification of all positions, then the samples were PCR cleaned using the Zymo 96-well DNA clean and concentrate kit, eluted in 10μL nuclease free water, pooled together in a low DNA-bind tube, and then DNA cleaned (Zymo) once more to further concentrate the pooled library.

The kinase domain variant library was digested with PstI-HF (NEB) and NdeI-HF (NEB) and cleaned up with the Zymo DNA clean and concentrate kit. Next, the two cloning vectors (pUC19_kozak-TPR-METΔEx14-IRES-eGFP and pUC19_kozak-TPR-MET-IRES-mCherry), were digested with PstI-HF(NEB) and NdeI-HF (NEB), phosphatase treated with rSAP (NEB), gel extracted to isolate the backbone, and DNA cleaned (Zymo). The variant library was ligated into each vector with a 1:3 (insert: vector) T4 ligation at 16°C overnight (NEB). Ligations were DNA cleaned (Zymo), eluted in 10uL of nuclease free water, and electroporated into 50μL MegaX 10 beta cells (Invitrogen). Transformations were then recovered in 1mL of SOC for 1hr at 37°C. Post recovery, 10μL cells were collected, serial diluted, plated at varying dilutions (1:100, 1:1k, 1:10k, 1:100k, 1:1M) to evaluate transformation efficiencies. The remainder of the transformation then propagated in 50mL LB and Carbyinacillin at 37°C to an OD of 0.5, and then midi-prepped (Zymo).

Amino acid variants were successfully synthesized by Twist Biosciences for all positions with the exception of 1194 and 1278. In addition, premature stop codons were not included in the synthesized Twist library. To include these missing positions and early stop control, we generated a “fill-in” library. For positions 1194 and 1278, a forward primer for each amino acid mutation and a single reverse primer was designed for inverse PCR. An early stop codon “fill-in” library was also generated to introduce one stop codon every 33 bases, evenly spaced throughout the gene. This resulted in one stop codon every 11 positions, or 24 total premature stops. Again, a single forward and reverse primer was designed for each stop codon mutation using inverse PCR. Mutations were introduced into wild type pUC19_kozak-TPR-METΔEx14-IRES-eGFP and pUC19_kozak-TPR-MET-IRES-mCherry with the following NEB Q5 High-Fidelity conditions per reaction: 10μL 5X Q5 buffer, 5ng template DNA, 2.5μL 10μM forward primer, 2.5μL 10μM reverse primer, 1μL 10mM dNTPs (2.5μM each), 0.5μL Q5 Polymerase, nuclease free water to a final volume of 50μL. The following thermocycler parameters were then applied: initial denaturation at 98°C for 30s, 10x cycles of denaturation at 98°C for 10s, annealing at 62°C for 30s, extension at 72°C for 4.4 min, and a final extension at 72°C for 10 min. A 1% agarose diagnostic gel was run with 2μL of each reaction to conform amplification. Then all PCR samples were pooled, DNA cleaned (Zymo) and eluted in 50μL nuclease free water, DPN1 digested to remove template (NEB), DNA cleaned again and eluted in 12μL nuclease free water, PNK treated (NEB), and blunt-end ligated at 16°C overnight with T4 ligase. Ligations were DNA cleaned the next morning (Zymo) and electroporated into MegaX 10 beta cells (Invitrogen). Transformations were recovered in 1mL of SOC for 1hr at 37°C, plated at varying dilutions to estimate transformation efficiencies, propagated in 50mL LB and Carbyinacillin at 37°C to an OD of 0.5, and then midi-prepped (Zymo). The Twist-synthesized library was then pooled together with the 1278, 1194, and premature stop “fill-in” libraries at equimolar concentrations to a total of 1ug of DNA.

7μg of each pooled library was restriction digested with MluI-HF (NEB) and MfeI-HF (NEB) to cut out variant libraries in the kozak-TPR-MET-IRES-mCherry and kozak-TPR-METΔEx14-IRES-eGFP backgrounds. Digests were gel extracted from pUC19 and DNA cleaned (Zymo). The empty, Puromycin resistant, retroviral expression vector MSCV (addgene) was also cut MluI-HF (NEB) and MfeI-HF (NEB), phosphatase treated with rSAP (NEB), and DNA cleaned. Isolated libraries were then ligated 1:1 (insert to vector) into the MSCV retroviral vector at 16°C overnight with T4 ligase. Ligations were then DNA cleaned (Zymo) and electroporated into ElectroMAX Stbl4 Competent Cells (Thermo Fisher). Transformations were recovered in 1mL of SOC for 1hr at 37°C and after recovery 10μL was serial diluted and plated to estimate transformation efficiencies, while the remainder was plated on bioassay dishes. Colonies were scraped from the bioassay dishes and midi-prepped for transfections (Zymo).

### Variant library introduction into Ba/F3

The MSCV_kozak-TPR-MET-IRES-mCherry and MSCV_kozak-TPR-METΔEx14-IRES-eGFP variant libraries were transfected into Plat-E cells for retroviral packaging using Lipofectamine3000 (Invitrogen). Two T-175 flasks of Plat-E cells were prepared for each library in the absence of blasticidin and puromycin 24hr prior to transfection such that they would be at 70–80% confluency at the time of transfection. On the day of transfection, each flask of Plat-E cells was gently washed with PBS to remove the culturing media, and replaced with 35mL Opti-MEM. For the transfection, Opti-MEM was brought to room temperature and two pairs of DNA Lo-bind 5mL tubes were prepared following manufacturer’s instructions for Lipofectamine3000 scaled to a T-175 format. A total of 46μg DNA was used to transfect the libraries and package virus in parallel: MSCV_TPR-MET-IRES-eGFP, MSCV_TPR-METΔEx14-IRES-mCherry. Each flask was incubated with the transfection reagents for 5hr at 37°C, 5% CO2; the transfection media was then replaced with 50mL OptiMEM, 5% FBS, 1x GlutaMax, and 2% Sodium Pyruvate (Gibco) for viral packaging. After 48 hr post-transfection, the viral supernatant was harvested, passed through a 0.45 μm filter to remove cell debris, then precipitated overnight with 1:4 Retro-X concentrator (TakaraBio) at 4°C, then pelleted at 380xg for 45 min at 4C, and resuspended in 5mL of sterile, cold PBS and stored at 4°C in 1mL aliquots until transduced into Ba/F3 cells.

The concentrated virus was titiered in Ba/F3 cells in a 6-well plate format. Cells were seeded at 1.0E5 cells/ml with 10 ng/ml IL-3 and 8μg/ml polybrene (Sigma-Aldrich). Virus was added to wells at 0, 10x, 20x, 40x dilutions to determine the proper volume for a transduction MOI of 0.1–0.3. Cells were spinfected at 250xg for 60 minutes at room temperature, then incubated for 48 hr. The viral titer was calculated from the percent of fluorescent cells and viral dilution.

For the DMS viral transduction, 6 million cells were spinfected at an MOI of 0.1, in triplicate for a total of 3 biological replicates for each library, and incubated post spinfection in a 15cm dish with 30mL Ba/F3 media and 10ng/L IL-3 for 48hr. Infected cells were then selected with 1μg/ml puromycin for a total of 4 days with fluorescence and cell counts tracked each day.

### DMS time point selection and sample preparation

After puromycin selection, all three biological replicates for both libraries, TPR-MET and TPR-METΔEx14, were washed free of puromycin and IL-3 with 3x PBS washes. A total of 6 million cells from each replicate was harvested and pelleted at 250xg to serve as the “time point 0” pre-selection sample (T0).

To begin selection of each replicate for each library, two sets of 15cm dishes were prepared with 2.0E5 cells/ml in 30mL 90% RPMI, 10% HI-FBS, 1% penicillin/streptomycin. One plate was kept free of IL-3 as the experimental IL-3 withdrawal condition, while the other plate was supplemented with 10ng/mL IL-3 to provide the control condition. Three time points post T0 were collected for each library replicate and condition for a total of four time points (T0, T1, T2, T3). Time points were harvested every 48hr across 7 days; 6 million cells were harvested for each condition and pelleted at 250xg for 5min; 2.0E5 cells/ml were split at every time point and maintained either in IL-3 or IL-3 withdrawal conditions.

The gDNA of each time point sample was isolated with the TakaraBio NucleoSpin Blood QuickPure kit the same day the cells were harvested. gDNA was eluted in a 50μl elution buffer using the high concentration and high yield elution manufacturer’s protocol. Immediately after gDNA was isolated, 5μg of gDNA was used for PCR amplification of the target MET KD gene to achieve the proper variant coverage. A 150μl PCR master mix was prepared for each sample using the TakaraBio PrimeStar GXL system according to the following recipe: 30μl 5X PrimeStar GXL buffer, 4.5μl 10μM forward primer (0.3uM final), 4.5μl 10μM reverse primer (0.3uM final), 5μg gDNA, 12μl 10mM dNTPs (2.5mM each NTP), 6μl GXL polymerase, nuclease free water to a final reaction volume of 150uL. The PCR master mix was split into three PCR tubes with 50μl volumes and amplified with the following thermocycler parameters: initial denaturation at 98°C for 30 s, followed by 24x cycles of denaturation at 98°C for 10 s, annealing at 60°C for 15 s, extension at 68°C for 14 s, and a final extension at 68°C for 1 min.

### Library preparation and deep sequencing

After all time points were selected, harvested, and PCR amplified, the target gene amplicon was isolated from gDNA by gel purification. The entire 150μl PCR reaction for each sample was mixed with 1X NEB Purple Loading Dye (6X stock) and run on a 0.8% agarose, 1X TBE gel, at 100 mA until there was clear ladder and band separation. The target amplicons were gel excised and purified with the Zymo Gel DNA Recovery kit. To remove excess agarose contamination, each sample was then further cleaned using the Zymo DNA Clean and Concentrator-5 kit. Amplicon DNA concentrations were then determined by Qubit dsDNA HS assay (Invitrogen).

Libraries were then prepared for deep sequencing using the Nextera XT DNA Library Prep kit in a 96-well plate format (Illumina). Manufacturer’s instructions were followed for each step: tagmentation, indexing and amplification, and clean up. Libraries were indexed using the IDT for Nextera Unique Dual Indexes Set C (Illumina). Then, indexed libraries were quantified using the Agilent TapeStation with HS D5000 screen tape (Agilent) and reagents (Agilent). DNA concentrations were further confirmed with a Qubit dsDNA HS assay. All samples were manually normalized and pooled at 10nM for MET and METΔEx14. The libraries were then paired-end sequenced (SP300) on two lanes of a NovaSeq6000.

### MET kinase domain variant analysis and scoring

Demultiplexed paired-end reads were received from the sequencing core and processed further using a snakemake-based pipeline previously developed (Macdonald et al., 2022; Mölder et al., 2021). Initial QC was performed via FastQC (Andrews and Others, 2010), and continued via aggregation of intermediate output statistics with MultiQC (Ewels et al., 2016). First, any remaining adapter sequences or contaminant sequences were removed with BBDuk. Next, overlap-based error-correction was employed with BBMerge, before being mapped to the reference sequence with BBMap (Bushnell, 2014). Variant counts from each mapped BAM file were then made with the GATK AnalyzeSaturationMutagenesis tool (Van der Auwera and O’Connor, 2020). The output of this tool was processed using a script to remove variants that were not in our initial library design and to prepare output in a format for further processing with Enrich2 using weighted-least squares with wild type normalization (Rubin et al., 2017). It was noted that Enrich2 produces unexpected scores when some variants are unobserved across replicates or go to zero over a single time course: to avoid this, our script also detects this and removes them in advance.

### MET and METΔEx14 mutational analysis

Raw Enrich2 scores were used for all comparative “activity score” measurements. gain-of-function and loss-of-function missense mutations were classified and calculated as ±2 SD from the mean activity score of the WT-synonymous distributions for MET and METΔEx14. For comparative analysis, propagation of error was calculated from the delta activity score and delta SE of each variant for MET and METΔEx14, and only variants with a SE difference lower than the activity score difference were used.

### Validation of variants in the MET and METΔEx14 receptor by western blot

HEK293 cells were transiently transfected with Lipofectamine3000 (Invitrogen) according to the manufacturer’s protocols. Cells were harvested 24 hours post transfection, lysed in buffer (50 mM Tris-HCl, pH 7.5, 150 mM NaCl, 2 mM EDTA, and 1 % w/v Triton X-100 supplemented with protease inhibitor tablets (Roche), 1 mM sodium fluoride and 1 mM sodium vanadate). Clarified lysates were incubated with G1 affinity resin (Genscript) overnight at 4°C. Resin was washed with lysis buffer (without inhibitors) and proteins eluted by addition of Laemmli sample reducing buffer. Proteins were separated by SDS-PAGE on a 4–15% gradient gel (BioRad) and transferred to PVDF membrane (Millipore). Membranes were probed with Flag (2368), MET pY1234/5 (3077) (Cell Signaling Technologies).

Human MET knockout HeLa cells were transiently transfected with Lipofectamine3000 (Invitrogen) according to the manufacturer’s protocols in a 6-well plate format. Post transfection (24hr), cells were washed with PBS (Gibco) (3x washes) to remove serum and transfection media, and replaced with DMEM (Gibco) in the absence of any additives. Cells were serum starved for 4 hr, then stimulated with 50 ng/ml HGF (PeproTech) for 15 min at 37°C, then immediately washed with cold PBS (3x washes), and maintained on ice. Cells were then lysed in buffer (50 mM Tris-HCl, pH 7.5, 150 mM NaCl, 2 mM EDTA, and 1 % w/v Triton X-100 supplemented with protease inhibitor tablets (Roche), 1 mM sodium fluoride, and 1 mM sodium vanadate) on ice. Clarified whole cell lysates were run on a 8–16% SDS-PAGE gel (BioRad) and transferred to a nitrocellulose membrane (BioRad). Membranes were probed with MET pY1234/5 (Cat# 3077), Met (8198), P-p44/42 MAPK Erk1/2 (T202/Y204) (4376), p44/42 MAPK Erk1/2 (4695), and β-Actin (4970) (Cell Signaling Technologies).

### Ba/F3 proliferation assay

Ba/F3 cells stably expressing TPR-MET, TPR-METΔEx14, and empty MSCV constructs were seeded at 2.5E4 cells/ml in triplicate in a 94-well, round bottom plate for each time point in the presence and absence of 10ng/ml IL-3. CellTiter-Glo reagent (Promega) was mixed at a 1:1 ratio with cells and luminescence was measured on a Veritas luminometer at 0, 48, and 96 hrs post-seeding. In this study, we use a modified nomenclature, where we refer to TPR-MET as the TPR-fusion of MET with the full-length juxtamembrane sequence and TPR-METΔEx14 as the TPR-fusion lacking exon 14. Cell numbers were determined from a Ba/F3 cell and ATP standard curve generated according to the manufacturer’s instructions. Data are presented as cell viability normalized to the fold change from the 0hr time point.

For IL-3 titrations, Ba/F3 cells stably expressing TPR-MET, TPR-METΔEx14, and empty MSCV constructs were 3x PBS washed and 5000 cells were seeded in a 94-well, round bottom plate. IL-3 was added to wells at 0–10ng/ml (0, 0.078, 0.16, 0.31, 1.3, 2.5, 5, 10 ng/ml). CellTiter-Glo reagent (Promega) was mixed at a 1:1 ratio with cells and luminescence was measured on a Veritas luminometer at 0, 24, and 48 hrs after seeding and IL-3 addition.

Cell numbers for all proliferation assays were determined from a Ba/F3 and ATP CellTiter-Glo standard curve generated according to the manufacturer’s instructions. Data are presented as cell viability normalized to the fold change from the 0hr time point.

### MET kinase domain structural ensemble and RTK structural comparisons

Structural visualization, mapping, and analysis was completed using PyMOL unless otherwise stated. All human MET (UniProtKB accession: P08581) kinase domain crystal structures currently available were downloaded from the PDB. All PDB structures were loaded and globally aligned to generate the kinase domain ensemble. Residue distances were calculated from alpha-carbon x,y,z coordinates and computationally analyzed. Raw PDB files were used to categorize structure features: resolution, construct boundaries, conformation, sequence features, mutations, and apo/holo states.

To choose representative active and inactive structures for score mapping and visualization, we generated an ensemble of 88 human MET kinase domain structures currently deposited in the PDB, and classified activity states based on alpha-carbon distances between catalytic site residues K1110, E1127, and F1223 (Modi and Dunbrack, 2019), with the majority of the MET KD structures in a “BLBplus” or “SRC-like” inactive conformation (Modi and Dunbrack, 2019). In study we refer to 3R7O, 3Q6W, 4IWD as as our representative “active” structures because they display classical active confirmation hallmarks (αC helix-in, K1110-E1127 salt bridge, DFG-in, solvent exposed A-loop despite being inhibited. Within the ensemble, there is only one ATP-bound structure (PDB 3DKC), which harbors A-loop Y1234F and Y1235D stabilizing mutations, and also displays an inactive conformation. Within the group of inactive structures, there are two main conformational species based on DFG/αC helix positioning and A-loop conformation: “BLBplus” and “BBAminus” (PDB 2G15 and PDB 5HTI represent the two species) (Modi and Dunbrack, 2019).

### RTK structural analysis and comparisons

Crystal structures of active and inactive human IR (PDB 4XLV, 4IBM), KIT (PDB 1PKG, 1T45), EPHA3 (PDB 2QO9), RET (PDB 2IVT, 2IVS), MET (PDB 3R7O, 2G15), and AXL (PDB 5UAB) were obtained through the PDB. RMSD was calculated and plotted with the Bio3D package in R for each kinase using the inactive structure as the reference.

For β5 positional comparison and Ramachandran analysis, PDB files were obtained for each kinase, and analyzed with the Bio3D package in R to attain the Phi and Psi angles of each residue. The general and proline contour data was obtained from Lovell et al., 2003 and plotted as an overlay with the specific kinase β5 residues aforementioned.

### Cancer and resistance mutation analysis

Cancer-associated missense mutations for the MET kinase domain was obtained from cBioPortal (NCBI ID: NM_000245). Resistance mutations were obtained from literature references (Duplaquet et al., 2018; Fernandes et al 2021; Saraon et al., 2021; Lu et al., 2017; Fujino et al., 2019).

### Sequence alignments

All human RTK protein sequences used in alignments were acquired from UniProt. Unless otherwise stated, alignments were done with MuscleWS using default parameters through JalView, and amino acids were colored according to physicochemical properties, or percent sequence identity where noted.

## Supplementary Material

Supplement 1

## References

[R1] Abella J. V., Peschard P., Naujokas M. A., Lin T., Saucier C., Urbé S., & Park M. (2005). Met/Hepatocyte Growth Factor Receptor Ubiquitination Suppresses Transformation and Is Required for Hrs Phosphorylation. Molecular and Cellular Biology, 25(21), 9632–9645. 10.1128/MCB.25.21.9632-9645.2005 16227611 PMC1265818

[R2] Ahler E., Register A. C., Chakraborty S., Fang L., Dieter E. M., Sitko K. A., Vidadala R. S. R., Trevillian B. M., Golkowski M., Gelman H., Stephany J. J., Rubin A. F., Merritt E. A., Fowler D. M., & Maly D. J. (2019). A Combined Approach Reveals a Regulatory Mechanism Coupling Src’s Kinase Activity, Localization, and Phosphotransferase-Independent Functions. Molecular Cell, 74(2), 393–408.e20. 10.1016/j.molcel.2019.02.003 30956043 PMC6474823

[R3] Andrews S., & Others. (2010). “FastQC: A Quality Control Tool for High Throughput Sequence Data.” Babraham Bioinformatics, Babraham Institute, Cambridge, United Kingdom. https://www.bioinformatics.babraham.ac.uk/projects/fastqc/

[R4] Azam M., Seeliger M. A., Gray N. S., Kuriyan J., & Daley G. Q. (2008). Activation of tyrosine kinases by mutation of the gatekeeper threonine. Nature Structural & Molecular Biology, 15(10), 1109–1118. 10.1038/nsmb.1486 PMC2575426 18794843

[R5] Bardelli A., Longati P., Gramaglia D., Basilico C., Tamagnone L., Giordano S., Ballinari D., Michieli P., & Comoglio P. M. (1998). Uncoupling signal transducers from oncogenic MET mutants abrogates cell transformation and inhibits invasive growth. Proceedings of the National Academy of Sciences, 95(24), 14379–14383. 10.1073/pnas.95.24.14379 PMC24381 9826708

[R6] Birchmeier C., Birchmeier W., Gherardi E., & Vande Woude G. F. (2003). Met, metastasis, motility and more. Nature Reviews Molecular Cell Biology, 4(12), 915–925. 10.1038/nrm1261 14685170

[R7] Brenan L., Andreev A., Cohen O., Pantel S., Kamburov A., Cacchiarelli D., Persky N. S., Zhu C., Bagul M., Goetz E. M., Burgin A. B., Garraway L. A., Getz G., Mikkelsen T. S., Piccioni F., Root D. E., & Johannessen C. M. (2016). Phenotypic Characterization of a Comprehensive Set of MAPK1/ERK2 Missense Mutants. Cell Reports, 17(4), 1171–1183. 10.1016/j.celrep.2016.09.061 27760319 PMC5120861

[R8] Bushnell B. 2014. “BBTools Software Package.”

[R9] Cabail M. Z., Li S., Lemmon E., Bowen M. E., Hubbard S. R., & Miller W. T. (2015). The insulin and IGF1 receptor kinase domains are functional dimers in the activated state. Nature Communications, 6(1), 6406. 10.1038/ncomms7406 PMC4758444 25758790

[R10] Cerami E., Gao J., Dogrusoz U., Gross B. E., Sumer S. O., Aksoy B. A., Jacobsen A., Byrne C. J., Heuer M. L., Larsson E., Antipin Y., Reva B., Goldberg A. P., Sander C., & Schultz N. (2012). The cBio Cancer Genomics Portal: An Open Platform for Exploring Multidimensional Cancer Genomics Data. Cancer Discovery, 2(5), 401–404. 10.1158/2159-8290.CD-12-0095 22588877 PMC3956037

[R11] Chakraborty S., Ahler E., Simon J. J., Fang L., Potter Z. E., Sitko K. A., Stephany J. J., Guttman M., Fowler D. M., & Maly D. J. (2021). Profiling of the drug resistance of thousands of Src tyrosine kinase mutants uncovers a regulatory network that couples autoinhibition to the dynamics of the catalytic domain [Preprint]. Biochemistry. 10.1101/2021.12.05.471322 PMC10902203 37683649

[R12] Chen H., Ma J., Li W., Eliseenkova A. V., Xu C., Neubert T. A., Miller W. T., & Mohammadi M. (2007). A Molecular Brake in the Kinase Hinge Region Regulates the Activity of Receptor Tyrosine Kinases. Molecular Cell, 27(5), 717–730. 10.1016/j.molcel.2007.06.028 17803937 PMC2094128

[R13] Chen L., Marsiglia W. M., Chen H., Katigbak J., Erdjument-Bromage H., Kemble D. J., Fu L., Ma J., Sun G., Zhang Y., Liang G., Neubert T. A., Li X., Traaseth N. J., & Mohammadi M. (2020). Molecular basis for receptor tyrosine kinase A-loop tyrosine transphosphorylation. Nature Chemical Biology, 16(3), Article 3. 10.1038/s41589-019-0455-7 PMC7040854 31959966

[R14] Chiara F., Michieli P., Pugliese L., & Comoglio P. M. (2003). Mutations in the met Oncogene Unveil a “Dual Switch” Mechanism Controlling Tyrosine Kinase Activity. Journal of Biological Chemistry, 278(31), 29352–29358. 10.1074/jbc.M302404200 12746450

[R15] Cooper C. S., Park M., Blair D. G., Tainsky M. A., Huebner K., Croce C. M., & Vande Woude G. F. (1984). Molecular cloning of a new transforming gene from a chemically transformed human cell line. Nature, 311(5981), 29–33. 10.1038/311029a0 6590967

[R16] Comoglio P. M., Trusolino L., & Boccaccio C. (2018). Known and novel roles of the MET oncogene in cancer: A coherent approach to targeted therapy. Nature Reviews Cancer, 18(6), Article 6. 10.1038/s41568-018-0002-y 29674709

[R17] Daley G. Q., & Baltimore D. (1988). Transformation of an interleukin 3-dependent hematopoietic cell line by the chronic myelogenous leukemia-specific P210bcr/abl protein. Proceedings of the National Academy of Sciences, 85(23), 9312–9316. 10.1073/pnas.85.23.9312 PMC282729 3143116

[R18] Dar A. C., & Shokat K. M. (2011). The Evolution of Protein Kinase Inhibitors from Antagonists to Agonists of Cellular Signaling. Annual Review of Biochemistry, 80(1), 769–795. 10.1146/annurev-biochem-090308-173656 21548788

[R19] Duplaquet L., Kherrouche Z., Baldacci S., Jamme P., Cortot A. B., Copin M.-C., & Tulasne D. (2018). The multiple paths towards MET receptor addiction in cancer. Oncogene, 37(24), 3200–3215. 10.1038/s41388-018-0185-4 29551767

[R20] Tovar Elizabeth A., & Graveel C. R. (2017). MET in human cancer: Germline and somatic mutations. Annals of Translational Medicine, 5(10), Article 10. 10.21037/atm.2017.03.64 PMC5451609 28603720

[R21] Ewels P., Magnusson M., Lundin S., & Käller M. (2016). MultiQC: Summarize analysis results for multiple tools and samples in a single report. Bioinformatics, 32(19), 3047–3048. 10.1093/bioinformatics/btw354 27312411 PMC5039924

[R22] Fernandes M., Hoggard B., Jamme P., Paget S., Truong M.-J., Grégoire V., Vinchent A., Descarpentries C., Morabito A., Stanislovas J., Farage E., Meneboo J.-P., Sebda S., Bouchekioua-Bouzaghou K., Nollet M., Humez S., Perera T., Fromme P., Grumolato L., … Kherrouche Z. (2023). MET exon 14 skipping mutation is a hepatocyte growth factor (HGF)-dependent oncogenic driver in vitro and in humanized HGF knock-in mice. Molecular Oncology. 10.1002/1878-0261.13397 PMC10620121 36799689

[R23] Fernandes M., Jamme P., Cortot A. B., Kherrouche Z., & Tulasne D. (2021). When the MET receptor kicks in to resist targeted therapies. Oncogene, 40(24), 4061–4078. 10.1038/s41388-021-01835-0 34031544

[R24] Ferracini R., Longati P., Naldini L., Vigna E., & Comoglio P. M. (1991). Identification of the major autophosphorylation site of the Met/hepatocyte growth factor receptor tyrosine kinase. Journal of Biological Chemistry, 266(29), 19558–19564. 10.1016/S0021-9258(18)55031-6 1655790

[R25] Fowler D. M., & Fields S. (2014). Deep mutational scanning: A new style of protein science. Nature Methods, 11(8), 801–807. 10.1038/nmeth.3027 25075907 PMC4410700

[R26] Frampton G. M., Ali S. M., Rosenzweig M., Chmielecki J., Lu X., Bauer T. M., Akimov M., Bufill J. A., Lee C., Jentz D., Hoover R., Ou S.-H. I., Salgia R., Brennan T., Chalmers Z. R., Jaeger S., Huang A., Elvin J. A., Erlich R., … Miller V. A. (2015). Activation of MET via Diverse Exon 14 Splicing Alterations Occurs in Multiple Tumor Types and Confers Clinical Sensitivity to MET Inhibitors. Cancer Discovery, 5(8), 850–859. 10.1158/2159-8290.CD-15-0285 25971938

[R27] Fujino T., Kobayashi Y., Suda K., Koga T., Nishino M., Ohara S., Chiba M., Shimoji M., Tomizawa K., Takemoto T., & Mitsudomi T. (2019). Sensitivity and Resistance of MET Exon 14 Mutations in Lung Cancer to Eight MET Tyrosine Kinase Inhibitors In Vitro. Journal of Thoracic Oncology, 14(10), 1753–1765. 10.1016/j.jtho.2019.06.023 31279006

[R28] Gajiwala K. S., Grodsky N., Bolaños B., Feng J., Ferre R., Timofeevski S., Xu M., Murray B. W., Johnson T. W., & Stewart A. (2017). The Axl kinase domain in complex with a macrocyclic inhibitor offers first structural insights into an active TAM receptor kinase. Journal of Biological Chemistry, 292(38), 15705–15716. 10.1074/jbc.M116.771485 28724631 PMC5612104

[R29] Gao J., Aksoy B. A., Dogrusoz U., Dresdner G., Gross B., Sumer S. O., Sun Y., Jacobsen A., Sinha R., Larsson E., Cerami E., Sander C., & Schultz N. (2013). Integrative Analysis of Complex Cancer Genomics and Clinical Profiles Using the cBioPortal. Science Signaling, 6(269), pl1–pl1. 10.1126/scisignal.2004088 23550210 PMC4160307

[R30] Gherardi E., Sandin S., Petoukhov M. V., Finch J., Youles M. E., Öfverstedt L.-G., Miguel R. N., Blundell T. L., Vande Woude G. F., Skoglund U., & Svergun D. I. (2006). Structural basis of hepatocyte growth factor/scatter factor and MET signalling. Proceedings of the National Academy of Sciences, 103(11), 4046–4051. 10.1073/pnas.0509040103 PMC1449643 16537482

[R31] Gherardi E., Youles M. E., Miguel R. N., Blundell T. L., Iamele L., Gough J., Bandyopadhyay A., Hartmann G., & Butler P. J. G. (2003). Functional map and domain structure of MET, the product of the c-met protooncogene and receptor for hepatocyte growth factor/scatter factor. Proceedings of the National Academy of Sciences, 100(21), 12039–12044. 10.1073/pnas.2034936100 PMC218709 14528000

[R32] Graziani A., Gramaglia D., Cantley L. C., & Comoglio P. M. (1991). The tyrosine-phosphorylated hepatocyte growth factor/scatter factor receptor associates with phosphatidylinositol 3-kinase. Journal of Biological Chemistry, 266(33), 22087–22090. 10.1016/S0021-9258(18)54536-1 1718989

[R33] Griffith J., Black J., Faerman C., Swenson L., Wynn M., Lu F., Lippke J., & Saxena K. (2004). The Structural Basis for Autoinhibition of FLT3 by the Juxtamembrane Domain. Molecular Cell, 13(2), 169–178. 10.1016/S1097-2765(03)00505-7 14759363

[R34] Haling J. R., Sudhamsu J., Yen I., Sideris S., Sandoval W., Phung W., Bravo B. J., Giannetti A. M., Peck A., Masselot A., Morales T., Smith D., Brandhuber B. J., Hymowitz S. G., & Malek S. (2014). Structure of the BRAF-MEK Complex Reveals a Kinase Activity Independent Role for BRAF in MAPK Signaling. Cancer Cell, 26(3), 402–413. 10.1016/j.ccr.2014.07.007 25155755

[R35] Hedger G., Sansom M. S. P., & Koldsø H. (2015). The juxtamembrane regions of human receptor tyrosine kinases exhibit conserved interaction sites with anionic lipids. Scientific Reports, 5(1), Article 1. 10.1038/srep09198 PMC4361843 25779975

[R36] Hobbs H. T., Shah N. H., Shoemaker S. R., Amacher J. F., Marqusee S., & Kuriyan J. (2022). Saturation mutagenesis of a predicted ancestral Syk-family kinase (p. 2022.04.24.489292). bioRxiv. 10.1101/2022.04.24.489292 PMC9601881 36173161

[R37] Hu J., Ahuja L. G., Meharena H. S., Kannan N., Kornev A. P., Taylor S. S., & Shaw A. S. (2015). Kinase Regulation by Hydrophobic Spine Assembly in Cancer. Molecular and Cellular Biology, 35(1), 264–276. 10.1128/MCB.00943-14 25348715 PMC4295384

[R38] Hubbard S. R. (2004). Juxtamembrane autoinhibition in receptor tyrosine kinases. Nature Reviews Molecular Cell Biology, 5(6), 464–471. 10.1038/nrm1399 15173825

[R39] Huse M., & Kuriyan J. (2002). The Conformational Plasticity of Protein Kinases. Cell, 109(3), 275–282. 10.1016/S0092-8674(02)00741-9 12015977

[R40] Jura N., Endres N. F., Engel K., Deindl S., Das R., Lamers M. H., Wemmer D. E., Zhang X., & Kuriyan J. (2009). Mechanism for Activation of the EGF Receptor Catalytic Domain by the Juxtamembrane Segment. Cell, 137(7), 1293–1307. 10.1016/j.cell.2009.04.025 19563760 PMC2814540

[R41] Kang J., Deng Q.-M., Feng W., Chen Z.-H., Su J.-W., Chen H.-J., Wang W., Zhang S., Wang Q., Chen Z., Zhong W.-Z., Xu C., & Yang J.-J. (2023). Response and acquired resistance to MET inhibitors in de novo MET fusion-positive advanced non-small cell lung cancer. Lung Cancer, 178, 66–74. 10.1016/j.lungcan.2023.01.017 36806896

[R42] Kato T. (2017). Biological roles of hepatocyte growth factor‑Met signaling from genetically modified animals (Review). Biomedical Reports, 7(6), 495–503. 10.3892/br.2017.1001 29188052 PMC5702962

[R43] Knowles P. P., Murray-Rust J., Kjær S., Scott R. P., Hanrahan S., Santoro M., Ibáñez C. F., & McDonald N. Q. (2006). Structure and Chemical Inhibition of the RET Tyrosine Kinase Domain. Journal of Biological Chemistry, 281(44), 33577–33587. 10.1074/jbc.M605604200 16928683

[R44] Koga T., Suda K., & Mitsudomi T. (2022). Utility of the Ba/F3 cell system for exploring on-target mechanisms of resistance to targeted therapies for lung cancer. Cancer Science, 113(3), 815–827. 10.1111/cas.15263 34997674 PMC8898722

[R45] Kong-Beltran M., Seshagiri S., Zha J., Zhu W., Bhawe K., Mendoza N., Holcomb T., Pujara K., Stinson J., Fu L., Severin C., Rangell L., Schwall R., Amler L., Wickramasinghe D., & Yauch R. (2006). Somatic Mutations Lead to an Oncogenic Deletion of Met in Lung Cancer. Cancer Research, 66(1), 283–289. 10.1158/0008-5472.CAN-05-2749 16397241

[R46] Kornev A. P., Haste N. M., Taylor S. S., & Ten Eyck L. F. (2006). Surface comparison of active and inactive protein kinases identifies a conserved activation mechanism. Proceedings of the National Academy of Sciences, 103(47), 17783–17788. 10.1073/pnas.0607656103 PMC1693824 17095602

[R47] Kornev A. P., Taylor S. S., & Ten Eyck L. F. (2008). A helix scaffold for the assembly of active protein kinases. Proceedings of the National Academy of Sciences, 105(38), 14377–14382. 10.1073/pnas.0807988105 PMC2533684 18787129

[R48] Lee B. J., & Shah N. P. (2017). Identification and characterization of activating ABL1 1b kinase mutations: Impact on sensitivity to ATP-competitive and allosteric ABL1 inhibitors. Leukemia, 31(5), 1096–1107. 10.1038/leu.2016.353 27890928

[R49] Lemmon M. A., & Schlessinger J. (2010). Cell Signaling by Receptor Tyrosine Kinases. Cell, 141(7), 1117–1134. 10.1016/j.cell.2010.06.011 20602996 PMC2914105

[R50] Levina A., Fleming K. D., Burke J. E., & Leonard T. A. (2022). Activation of the essential kinase PDK1 by phosphoinositide-driven trans-autophosphorylation. Nature Communications, 13(1), 1874. 10.1038/s41467-022-29368-4 PMC8986801 35387990

[R51] Li S., Covino N. D., Stein E. G., Till J. H., & Hubbard S. R. (2003). Structural and Biochemical Evidence for an Autoinhibitory Role for Tyrosine 984 in the Juxtamembrane Region of the Insulin Receptor. Journal of Biological Chemistry, 278(28), 26007–26014. 10.1074/jbc.M302425200 12707268

[R52] Linossi E. M., Estevam G. O., Oshima M., Fraser J. S., Collisson E. A., & Jura N. (2021). State of the structure address on MET receptor activation by HGF. Biochemical Society Transactions, 49(2), 645–661. 10.1042/BST20200394 33860789 PMC8711257

[R53] Liu J., Shen L., Qian Y., Liu Y., Su M., & Yi L. (2022). Durable response to crizotinib in an advanced lung adenocarcinoma patient harboring rare CD47-MET fusion: A case report. Translational Cancer Research, 11(8). 10.21037/tcr-22-141 PMC9459510 36093550

[R54] Longati P., Bardelli A., Ponzetto C., Naldini L., & Comoglio P. M. (1994). Tyrosines1234–1235 are critical for activation of the tyrosine kinase encoded by the MET proto-oncogene (HGF receptor). Oncogene, 9(1), 49–57. 8302603

[R55] Lovell S. C., Davis I. W., Arendall III W. B., de Bakker P. I. W., Word J. M., Prisant M. G., Richardson J. S., & Richardson D. C. (2003). Structure validation by Cα geometry: ϕ,ψ and Cβ deviation. Proteins: Structure, Function, and Bioinformatics, 50(3), 437–450. 10.1002/prot.10286 12557186

[R56] Lu X., Peled N., Greer J., Wu W., Choi P., Berger A. H., Wong S., Jen K.-Y., Seo Y., Hann B., Brooks A., Meyerson M., & Collisson E. A. (2017). MET Exon 14 Mutation Encodes an Actionable Therapeutic Target in Lung Adenocarcinoma. Cancer Research, 77(16), 4498–4505. 10.1158/0008-5472.CAN-16-1944 28522754 PMC6004099

[R57] Ma P. C., Kijima T., Maulik G., Fox E. A., Sattler M., Griffin J. D., Johnson B. E., & Salgia R. (2003). c-MET Mutational Analysis in Small Cell Lung Cancer: Novel Juxtamembrane Domain Mutations Regulating Cytoskeletal Functions1. Cancer Research, 63(19), 6272–6281. 14559814

[R58] Macdonald C. B., Nedrud D., Grimes P. R., Trinidad D., Fraser J. S., & Coyote-Maestas W. (2023). DIMPLE: Deep insertion, deletion, and missense mutation libraries for exploring protein variation in evolution, disease, and biology. Genome Biology, 24(1), 36. 10.1186/s13059-023-02880-6 36829241 PMC9951526

[R59] Mak H. H. L., Peschard P., Lin T., Naujokas M. A., Zuo D., & Park M. (2007). Oncogenic activation of the Met receptor tyrosine kinase fusion protein, Tpr–Met, involves exclusion from the endocytic degradative pathway. Oncogene, 26(51), 7213–7221. 10.1038/sj.onc.1210522 17533376

[R60] McClendon C. L., Kornev A. P., Gilson M. K., & Taylor S. S. (2014). Dynamic architecture of a protein kinase. Proceedings of the National Academy of Sciences, 111(43), E4623–E4631. 10.1073/pnas.1418402111 PMC4217441 25319261

[R61] Meharena H. S., Chang P., Keshwani M. M., Oruganty K., Nene A. K., Kannan N., Taylor S. S., & Kornev A. P. (2013). Deciphering the Structural Basis of Eukaryotic Protein Kinase Regulation. PLoS Biology, 11(10), e1001680. 10.1371/journal.pbio.1001680 24143133 PMC3797032

[R62] Melnick J. S., Janes J., Kim S., Chang J. Y., Sipes D. G., Gunderson D., Jarnes L., Matzen J. T., Garcia M. E., Hood T. L., Beigi R., Xia G., Harig R. A., Asatryan H., Yan S. F., Zhou Y., Gu X.-J., Saadat A., Zhou V., … Caldwell J. S. (2006). An efficient rapid system for profiling the cellular activities of molecular libraries. Proceedings of the National Academy of Sciences, 103(9), 3153–3158. 10.1073/pnas.0511292103 PMC1413928 16492761

[R63] Modi V., & Dunbrack R. L. (2019). Defining a new nomenclature for the structures of active and inactive kinases. Proceedings of the National Academy of Sciences, 116(14), 6818–6827. 10.1073/pnas.1814279116 PMC6452665 30867294

[R64] Mölder F., Jablonski K. P., Letcher B., Hall M. B., Tomkins-Tinch C. H., Sochat V., Forster J., Lee S., Twardziok S. O., Kanitz A., Wilm A., Holtgrewe M., Rahmann S., Nahnsen S., & Köster J. (2021). Sustainable data analysis with Snakemake. F1000Research, 10, 33. 10.12688/f1000research.29032.2 34035898 PMC8114187

[R65] Naldini L., Vigna E., Ferracini R., Longati P., Gandino L., Prat M., & Comoglio P. M. (1991). The tyrosine kinase encoded by the MET proto-oncogene is activated by autophosphorylation. Molecular and Cellular Biology, 11(4), 1793–1803. 10.1128/mcb.11.4.1793-1803.1991 2005882 PMC359847

[R66] Pal K., Bandyopadhyay A., Zhou X. E., Xu Q., Marciano D. P., Brunzelle J. S., Yerrum S., Griffin P. R., Vande Woude G., Melcher K., & Xu H. E. (2017). Structural Basis of TPR-Mediated Oligomerization and Activation of Oncogenic Fusion Kinases. Structure, 25(6), 867–877.e3. 10.1016/j.str.2017.04.015 28528776 PMC5507626

[R67] Park M., Dean M., Cooper C. S., Schmidt M., O’Brien S. J., Blair D. G., & Vande Woude G. F. (1986). Mechanism of met oncogene activation. Cell, 45(6), 895–904. 10.1016/0092-8674(86)90564-7 2423252

[R68] Persky N. S., Hernandez D., Do Carmo M., Brenan L., Cohen O., Kitajima S., Nayar U., Walker A., Pantel S., Lee Y., Cordova J., Sathappa M., Zhu C., Hayes T. K., Ram P., Pancholi P., Mikkelsen T. S., Barbie D. A., Yang X., … Johannessen C. M. (2020). Defining the landscape of ATP-competitive inhibitor resistance residues in protein kinases. Nature Structural & Molecular Biology, 27(1), 92–104. 10.1038/s41594-019-0358-z 31925410

[R69] Peschard P., Fournier T. M., Lamorte L., Naujokas M. A., Band H., Langdon W. Y., & Park M. (2001). Mutation of the c-Cbl TKB Domain Binding Site on the Met Receptor Tyrosine Kinase Converts It into a Transforming Protein. Molecular Cell, 8(5), 995–1004. 10.1016/S1097-2765(01)00378-1 11741535

[R70] Petrelli A., Gilestro G. F., Lanzardo S., Comoglio P. M., Migone N., & Giordano S. (2002). The endophilin–CIN85–Cbl complex mediates ligand-dependent downregulation of c-Met. Nature, 416(6877), 187–190. 10.1038/416187a 11894096

[R71] Petrini I. (2015). Biology of MET: A double life between normal tissue repair and tumor progression. Annals of Translational Medicine, 3(6), Article 6. 10.3978/j.issn.2305-5839.2015.03.58 PMC4416948 25992381

[R72] Ponzetto C., Bardelli A., Zhen Z., Maina F., dalla Zonca P., Giordano S., Graziani A., Panayotou G., & Comoglio P. M. (1994). A multifunctional docking site mediates signaling and transformation by the hepatocyte growth factor/scatter factor receptor family. Cell, 77(2), 261–271. 10.1016/0092-8674(94)90318-2 7513258

[R73] Rettenmaier T. J., Sadowsky J. D., Thomsen N. D., Chen S. C., Doak A. K., Arkin M. R., & Wells J. A. (2014). A small-molecule mimic of a peptide docking motif inhibits the protein kinase PDK1. Proceedings of the National Academy of Sciences, 111(52), 18590–18595. 10.1073/pnas.1415365112 PMC4284534 25518860

[R74] Rodrigues G. A., & Park M. (1993). Dimerization mediated through a leucine zipper activates the oncogenic potential of the met receptor tyrosine kinase. Molecular and Cellular Biology, 13(11), 6711–6722. 8413267 10.1128/mcb.13.11.6711-6722.1993 PMC364734

[R75] Rubin A. F., Gelman H., Lucas N., Bajjalieh S. M., Papenfuss A. T., Speed T. P., & Fowler D. M. (2017). A statistical framework for analyzing deep mutational scanning data. Genome Biology, 18(1), 150. 10.1186/s13059-017-1272-5 28784151 PMC5547491

[R76] Saraon P., Pathmanathan S., Snider J., Lyakisheva A., Wong V., & Stagljar I. (2021). Receptor tyrosine kinases and cancer: Oncogenic mechanisms and therapeutic approaches. Oncogene, 40(24), 4079–4093. 10.1038/s41388-021-01841-2 34079087

[R77] Schiering N., Knapp S., Marconi M., Flocco M. M., Cui J., Perego R., Rusconi L., & Cristiani C. (2003). Crystal structure of the tyrosine kinase domain of the hepatocyte growth factor receptor c-Met and its complex with the microbial alkaloid K-252a. Proceedings of the National Academy of Sciences, 100(22), 12654–12659. 10.1073/pnas.1734128100 PMC240673 14559966

[R78] Sun D., Wu W., Wang L., Qu J., Han Q., Wang H., Song S., Liu N., Wang Y., & Hou H. (2023). Identification of MET fusions as novel therapeutic targets sensitive to MET inhibitors in lung cancer. Journal of Translational Medicine, 21(1), 150. 10.1186/s12967-023-03999-7 36829199 PMC9960416

[R79] Taylor S. S., & Kornev A. P. (2011). Protein kinases: Evolution of dynamic regulatory proteins. Trends in Biochemical Sciences, 36(2), 65–77. 10.1016/j.tibs.2010.09.006 20971646 PMC3084033

[R80] Till J. H., Becerra M., Watty A., Lu Y., Ma Y., Neubert T. A., Burden S. J., & Hubbard S. R. (2002). Crystal Structure of the MuSK Tyrosine Kinase: Insights into Receptor Autoregulation. Structure, 10(9), 1187–1196. 10.1016/S0969-2126(02)00814-6 12220490

[R81] Trusolino L., Bertotti A., & Comoglio P. M. (2010). MET signalling: Principles and functions in development, organ regeneration and cancer. Nature Reviews Molecular Cell Biology, 11(12), Article 12. 10.1038/nrm3012 21102609

[R82] Ung P. M.-U., Rahman R., & Schlessinger A. (2018). Redefining the Protein Kinase Conformational Space with Machine Learning. Cell Chemical Biology, 25(7), 916–924.e2. 10.1016/j.chembiol.2018.05.002 29861272 PMC6054563

[R83] Van der Auwera G. A., & O’Connor B. D. (2020). Genomics in the cloud: using Docker, GATK, and WDL in Terra. O’Reilly Media.

[R84] Vigna E., Gramaglia D., Longati P., Bardelli A., & Comoglio P. M. (1999). Loss of the exon encoding the juxtamembrane domain is essential for the oncogenic activation of TPR-MET. Oncogene, 18(29), 4275–4281. 10.1038/sj.onc.1202791 10435641

[R85] Wang W., Marimuthu A., Tsai J., Kumar A., Krupka H. I., Zhang C., Powell B., Suzuki Y., Nguyen H., Tabrizizad M., Luu C., & West B. L. (2006). Structural characterization of autoinhibited c-Met kinase produced by coexpression in bacteria with phosphatase. Proceedings of the National Academy of Sciences, 103(10), 3563–3568. 10.1073/pnas.0600048103 PMC1450123 16537444

[R86] Warmuth M., Kim S., Gu X., Xia G., & Adrián F. (2007). Ba/F3 cells and their use in kinase drug discovery. Current Opinion in Oncology, 19(1), 55–60. 10.1097/CCO.0b013e328011a25f 17133113

[R87] Weidner K. M., Di Cesare S., Sachs M., Brinkmann V., Behrens J., & Birchmeier W. (1996). Interaction between Gab1 and the c-Met receptor tyrosine kinase is responsible for epithelial morphogenesis. Nature, 384(6605), Article 6605. 10.1038/384173a0 8906793

[R88] Weingartner K. A., Tran T., Tripp K. W., & Kavran J. M. (2023). Dimerization and autophosphorylation of the MST family of kinases are controlled by the same set of residues (p. 2023.03.09.531926). bioRxiv. 10.1101/2023.03.09.531926 PMC10500444 37459121

[R89] Wiesner S., Wybenga-Groot L. E., Warner N., Lin H., Pawson T., Forman-Kay J. D., & Sicheri F. (2006). A change in conformational dynamics underlies the activation of Eph receptor tyrosine kinases. The EMBO Journal, 25(19), 4686–4696. 10.1038/sj.emboj.7601315 16977320 PMC1589994

[R90] Wybenga-Groot L. E., Baskin B., Ong S. H., Tong J., Pawson T., & Sicheri F. (2001). Structural Basis for Autoinhibition of the EphB2 Receptor Tyrosine Kinase by the Unphosphorylated Juxtamembrane Region. Cell, 106(6), 745–757. 10.1016/S0092-8674(01)00496-2 11572780

[R91] Yeung W., Ruan Z., & Kannan N. (2020). Emerging roles of the αC-β4 loop in protein kinase structure, function, evolution, and disease. IUBMB Life, 72(6), 1189–1202. 10.1002/iub.2253 32101380 PMC8375298

[R92] Zhang X., Gureasko J., Shen K., Cole P. A., & Kuriyan J. (2006). An Allosteric Mechanism for Activation of the Kinase Domain of Epidermal Growth Factor Receptor. Cell, 125(6), 1137–1149. 10.1016/j.cell.2006.05.013 16777603

