# Supplementary material for: Conserved regulatory motifs in the juxtamembrane domain and kinase N-lobe revealed through deep mutational scanning of the MET receptor tyrosine kinase domain
Source: bioRxiv. 2024 May 6:2023.08.03.551866. Originally published 2023 Aug 3. Preprint. [Version 3] doi: 10.1101/2023.08.03.551866 (PMC10418267; doi:10.1101/2023.08.03.551866)
Supplement: Supplement 1 [file NIHPP2023.08.03.551866v3-supplement-1.pdf]

bioRxiv preprint doi: https://doi.org/10.1101/2023.08.03.551866; this version posted May 7, 2024. The copyright holder for this preprint (which was not certified by peer review) is the author/funder, who has granted bioRxiv a license to display the preprint in perpetuity. It is made available under aCC-BY 4.0 International license.

Supplemental information

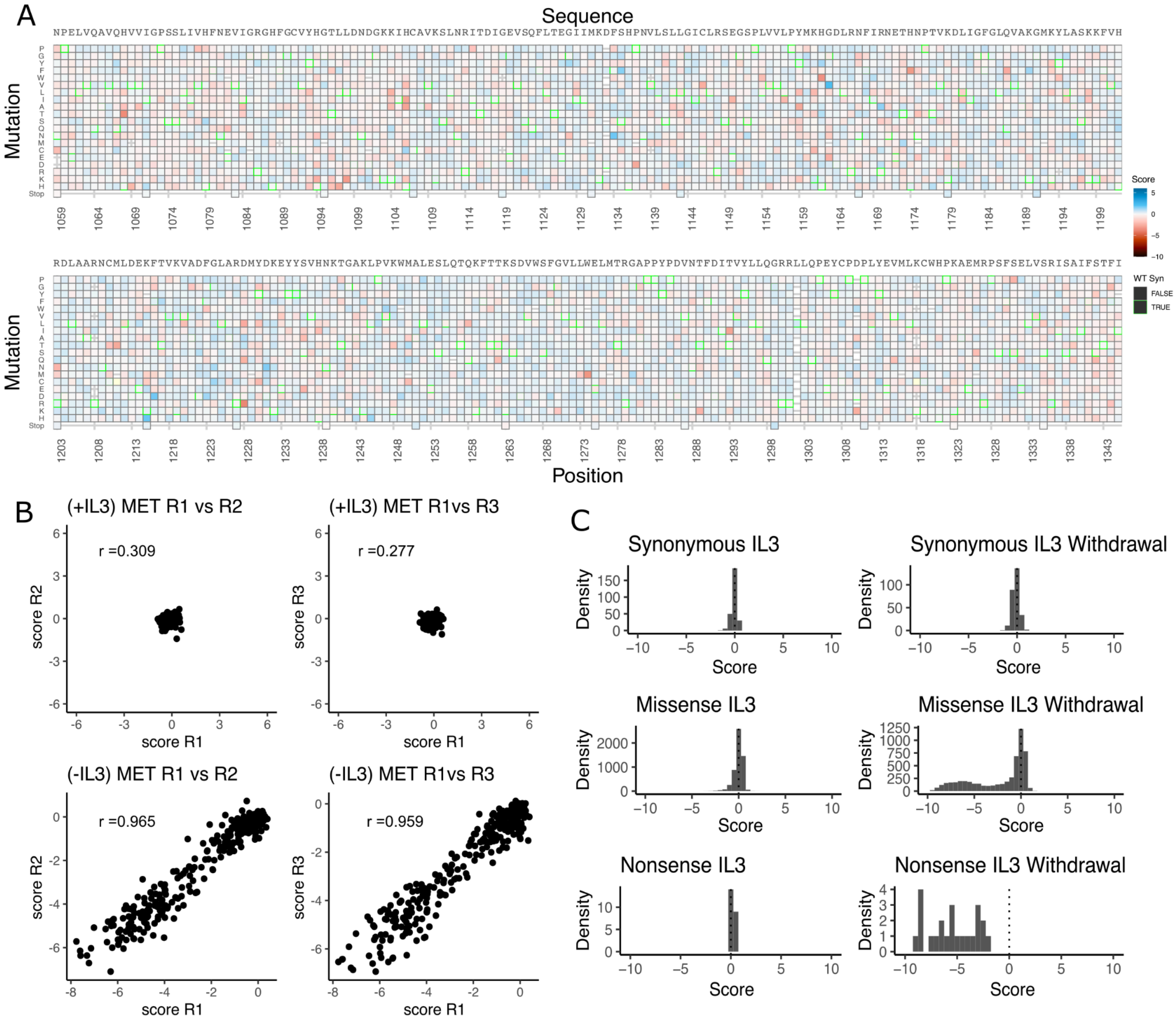

Supplemental Data Figure 1. Validation of the MET kinase domain saturation mutagenesis library in IL-3 and IL-3 withdrawal selections. (A) Heatmap of TPR-MET kinase domain variants in the full-length juxtamembrane background for the IL-3 control condition that was run in parallel to the IL-3 withdrawal selection. (B) Replicate correlation analysis for both IL-3 and IL-3 withdrawal conditions, where the mean score for each position was plotted against the replicate value. Pearson's correlation score is reported in each respective graph. (C) Distributions of synonymous, missense, and nonsense mutations for IL-3 and IL-3 withdrawal conditions.

bioRxiv preprint doi: https://doi.org/10.1101/2023.08.03.551866; this version posted May 7, 2024. The copyright holder for this preprint (which was not certified by peer review) is the author/funder, who has granted bioRxiv a license to display the preprint in perpetuity. It is made available under aCC-BY 4.0 International license.

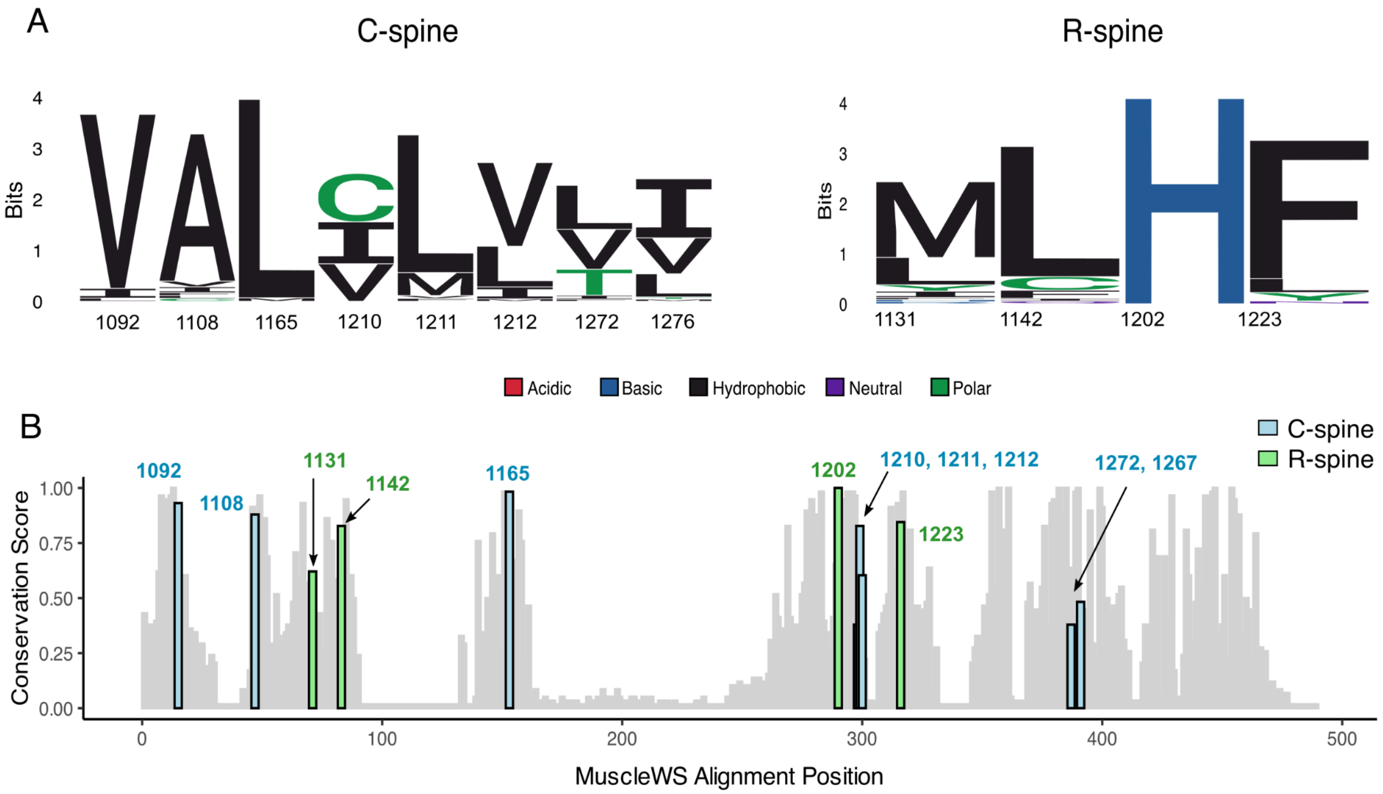

Supplemental Data Figure 2. Analysis of RTK R- and C-spine protein sequence conservation. (A) Logo diagram representing the amino acid conservation of the C- and R-spine across all RTK kinase domain protein sequences, aligned by MuscleWS. (B) Conservation score of residues at the corresponding MET C- and R-spine positions for all RTK kinase domain protein sequences, aligned by MuscleWS and scored through the bio3D package "conserv" function using a BLOSUM62 scoring matrix.

bioRxiv preprint doi: https://doi.org/10.1101/2023.08.03.551866; this version posted May 7, 2024. The copyright holder for this preprint (which was not certified by peer review) is the author/funder, who has granted bioRxiv a license to display the preprint in perpetuity. It is made available under aCC-BY 4.0 International license.

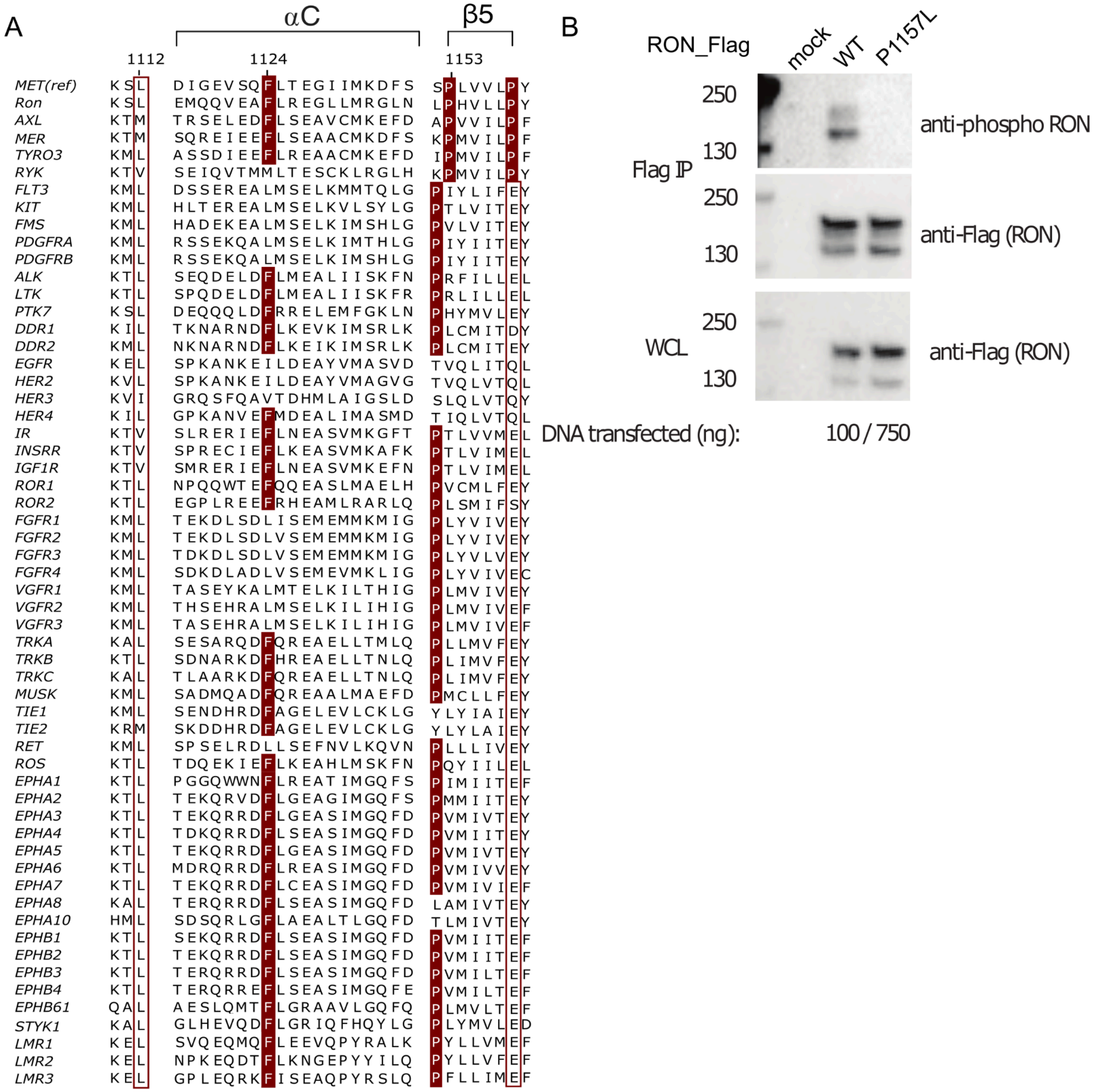

Supplemental Data Figure 3. RTK β5-turn site sequence analysis and validation in RON. (A) Protein sequence alignment of human RTK kinase domains with conservation highlighted for residues corresponding to MET positions 1112, 1124, 1153, and 1158. (B) FLAG-IP Western blot of RON with a P1157L mutation at the corresponding β5-turn site in the MET receptor, expressed for 24hr in HEK293 cells with input transfection DNA concentrations for wild type RON and P1157L constructs.

bioRxiv preprint doi: https://doi.org/10.1101/2023.08.03.551866; this version posted May 7, 2024. The copyright holder for this preprint (which was not certified by peer review) is the author/funder, who has granted bioRxiv a license to display the preprint in perpetuity. It is made available under aCC-BY 4.0 International license.

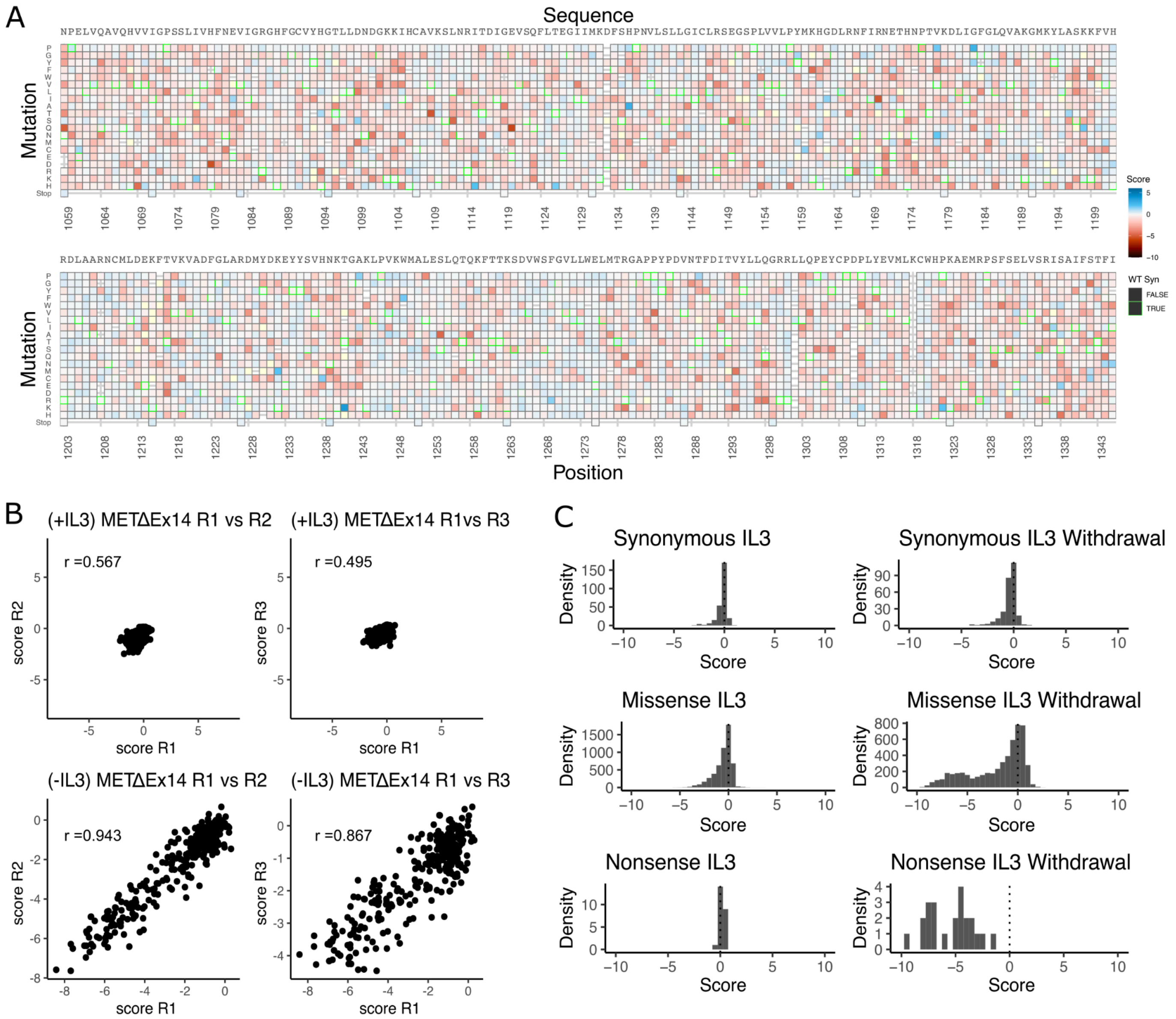

Supplemental Data Figure 4. Validation of the METΔEx14 saturation mutagenesis library in IL-3 and IL-3 withdrawal selections. (A) Heatmap of TPR-METΔEx14 kinase domain variants in the IL-3 control condition that was run in parallel to the IL-3 withdrawal selection. (B) Replicate correlation analysis for both IL-3 and IL-3 withdrawal, where the mean score for each position was plotted against the replicate value. Pearson's correlation score is reported in each respective graph. (C) Distributions of synonymous, missense, and nonsense mutations for IL-3 and IL-3 withdrawal conditions.

bioRxiv preprint doi: https://doi.org/10.1101/2023.08.03.551866; this version posted May 7, 2024. The copyright holder for this preprint (which was not certified by peer review) is the author/funder, who has granted bioRxiv a license to display the preprint in perpetuity. It is made available under aCC-BY 4.0 International license.

bioRxiv preprint doi: https://doi.org/10.1101/2023.08.03.551866; this version posted May 7, 2024. The copyright holder for this preprint (which was not certified by peer review) is the author/funder, who has granted bioRxiv a license to display the preprint in perpetuity. It is made available under aCC-BY 4.0 International license.

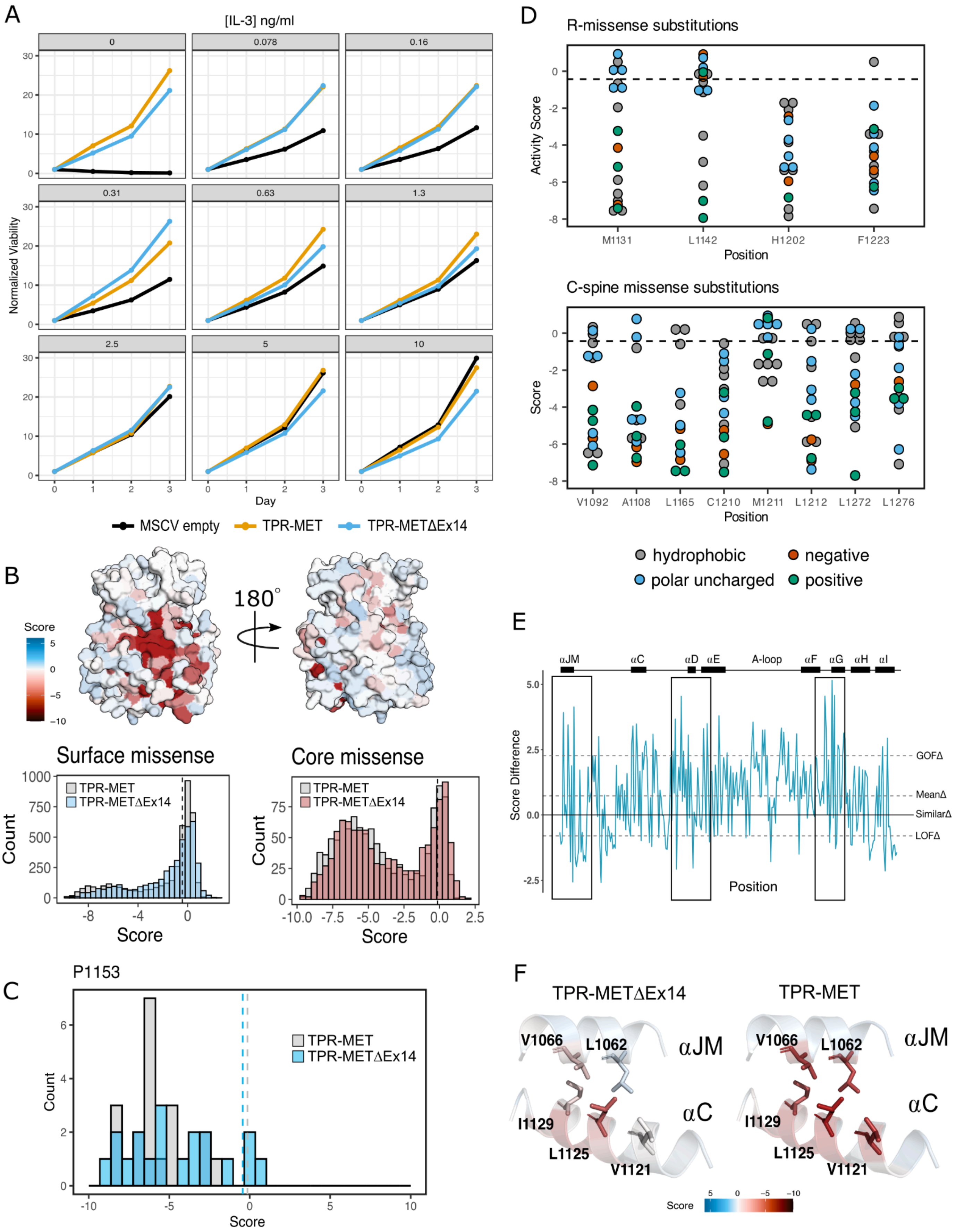

bioRxiv preprint doi: https://doi.org/10.1101/2023.08.03.551866; this version posted May 7, 2024. The copyright holder for this preprint (which was not certified by peer review) is the author/funder, who has granted bioRxiv a license to display the preprint in perpetuity. It is made available under aCC-BY 4.0 International license.

Supplemental Data Figure 5. Comparative analysis of the TPR-METΔEx14 and TPR-MET mutational landscapes. (A) IL-3 titration proliferation assay comparing Ba/F3 cells stably expressing TPR-MET, TPR-METΔEx14, and MSCV empty constructs over the course of 3 days at an IL-3 concentration range of 0, 0.078, 0.16, 0.31, 1.3, 2.5, 5, 10 ng/ml. Cell viability was normalized to day 0 for each condition. (B) Surface representation of TPR-METΔEx14 average mutational scores mapped on a TPR-MET kinase domain structure (PDB 3R7O). Synonymous and nonsense mutations were left out of the averaging and surface representation. Residues at the N- and C-term that were not screened, but modeled in the crystal structure are in white and not considered in the averaging and mapping. Comparison of surface and core residues scores distributions for TPR-METΔEx14, overlayed with the distributions for TPR-MET. A vertical dashed line in both graphs represents the mean score of WT-synonymous mutations. (C) Mutational distributions of P1153 variants for TPR-METΔEx14 (blue) and MET (gray). Dashed lines represent the mean of the WT-synonymous distribution for each library. (D) Mutation scores and physiochemistry of variants shown for each residue position of the R- and C- spine of TPR-METΔEx14. (E) Mean difference plot for TPR-METΔEx14 and TPR-MET at each screened position. (E) Average score of mutations at the hydrophobic interface of αJM and αC for both TPR-METΔEx14 and TPR-MET.

bioRxiv preprint doi: https://doi.org/10.1101/2023.08.03.551866; this version posted May 7, 2024. The copyright holder for this preprint (which was not certified by peer review) is the author/funder, who has granted bioRxiv a license to display the preprint in perpetuity. It is made available under aCC-BY 4.0 International license.

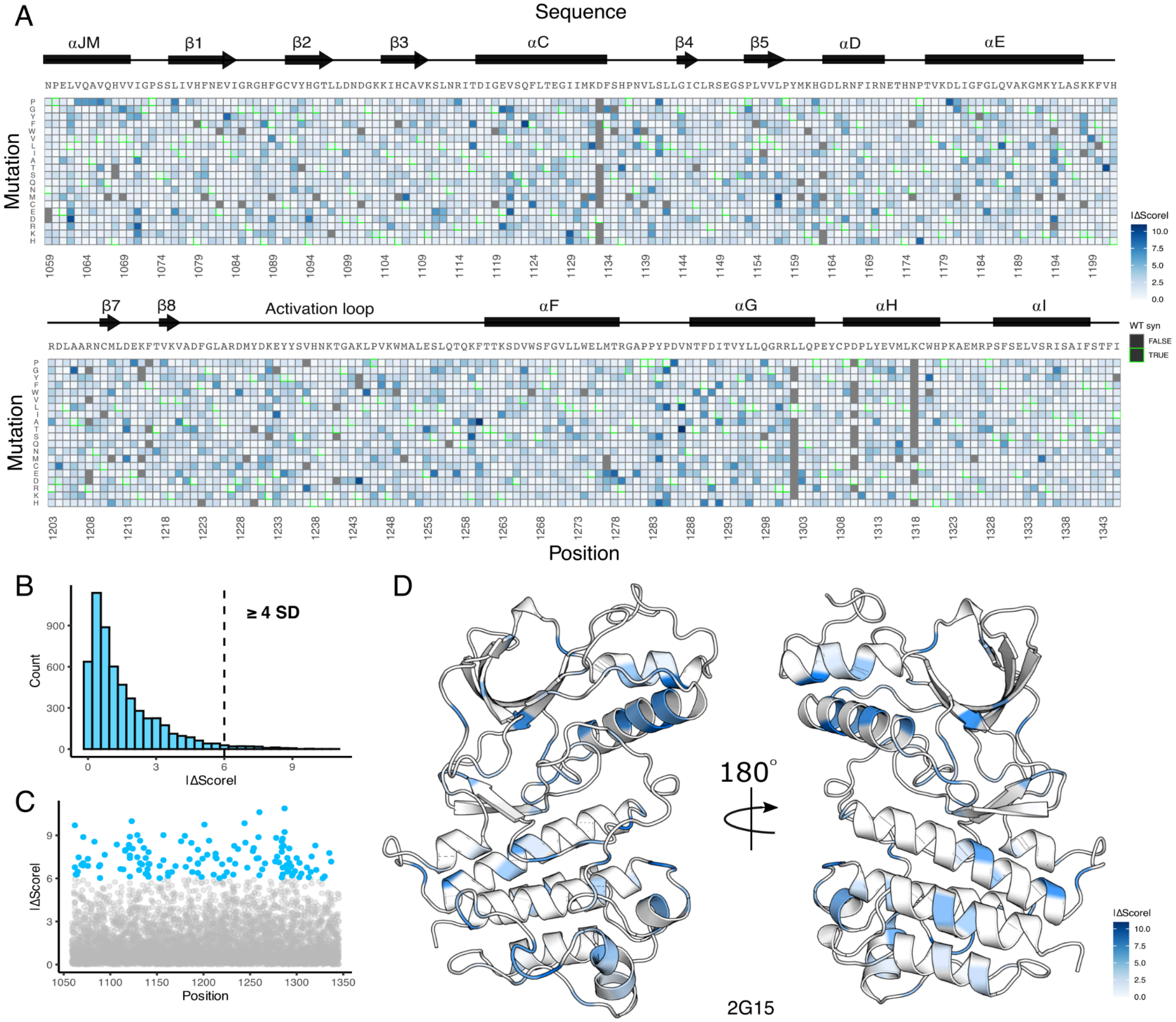

Supplemental Data Figure 6. Landscape of fitness and gain-of-function mutational differences between METΔEx14 and MET kinase domain libraries. (A) Heatmap showing the absolute difference between TPR-METΔEx14 and TPR-MET kinase domain mutation fitness scores. Mutations missing values for either one or both libraries are denoted in gray. (B) Distribution of the absolute score difference (|ΔScore|) between TPR-METΔEx14 and TPR-MET with a dashed line at the boundary for score differences above or equal to 4 standard deviations (SD) from the average |ΔScore|. (C-D) Scatter plot of all fitness score differences, with the specific mutations above a difference of 4 SD highlighted in blue, and further structurally mapped on a representative structure (PDB 2G15).

bioRxiv preprint doi: https://doi.org/10.1101/2023.08.03.551866; this version posted May 7, 2024. The copyright holder for this preprint (which was not certified by peer review) is the author/funder, who has granted bioRxiv a license to display the preprint in perpetuity. It is made available under aCC-BY 4.0 International license.

bioRxiv preprint doi: https://doi.org/10.1101/2023.08.03.551866; this version posted May 7, 2024. The copyright holder for this preprint (which was not certified by peer review) is the author/funder, who has granted bioRxiv a license to display the preprint in perpetuity. It is made available under aCC-BY 4.0 International license.

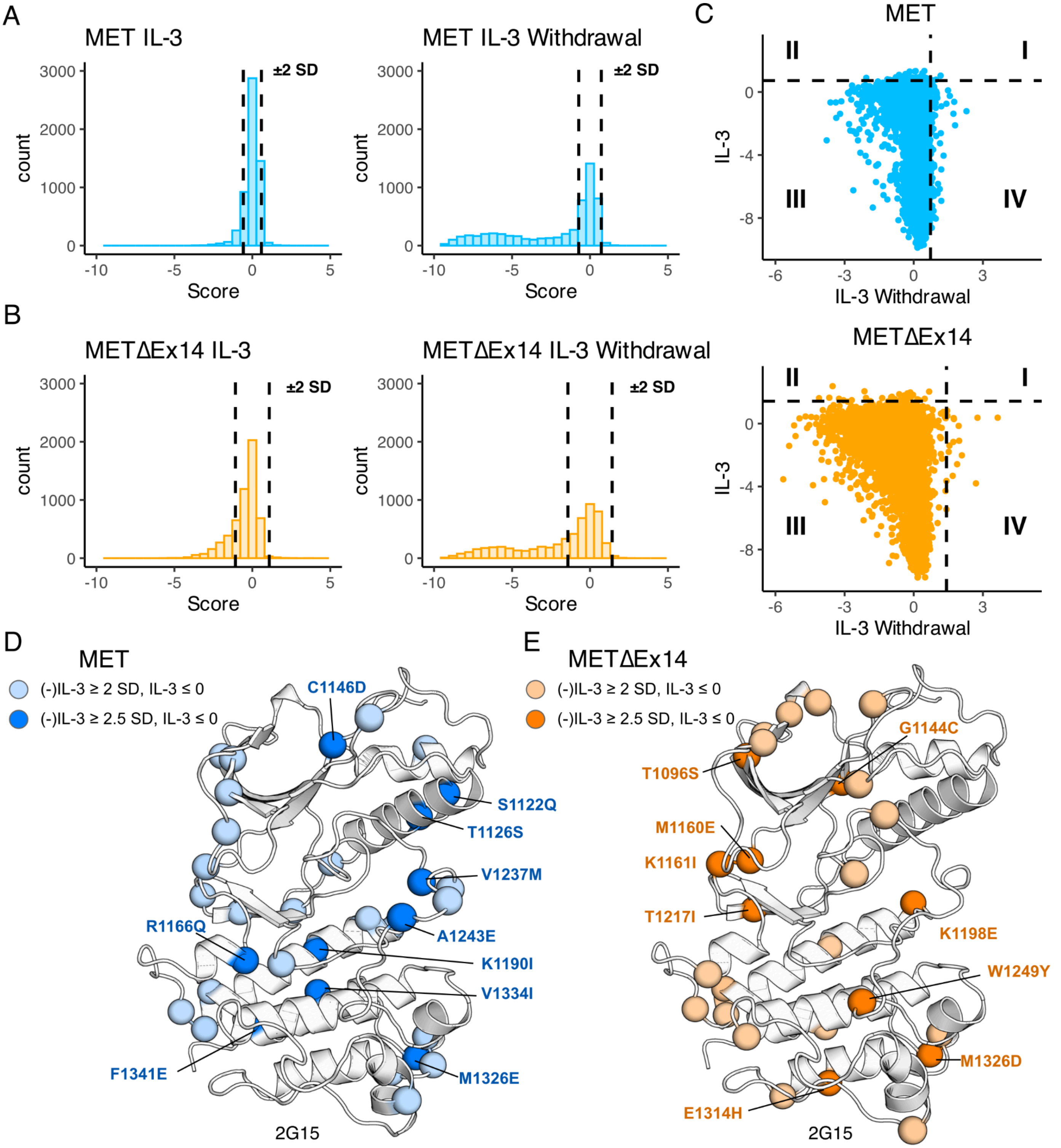

Supplemental Data Figure 7. Statistical analysis and classification of gain-of-function mutations across libraries. (A-B) Distribution of all mutations for TPR-MET and TPR-METΔEx14 kinase domain libraries with vertical dashed lines showing the ± 2 SD threshold from the wild type synonymous average. Mutations with fitness scores outside of these boundaries pass the initial filter for classification as gain-of-function (GOF) or loss-of-function (LOF). (C) Fitness score scatterplots for mutations under IL-3 withdrawal selection versus IL-3 maintenance for the TPR-MET and TPR-METΔEx14 kinase domain, with the +2 SD threshold displayed as a dashed line for both conditions, splitting the plot into four quadrants. Data points within quadrant IV are representative of mutations that have high fitness under IL-3 withdrawal selective pressure, that are otherwise null or loss-of-function in the absence of selection. (D-E) Statistically

bioRxiv preprint doi: https://doi.org/10.1101/2023.08.03.551866; this version posted May 7, 2024. The copyright holder for this preprint (which was not certified by peer review) is the author/funder, who has granted bioRxiv a license to display the preprint in perpetuity. It is made available under aCC-BY 4.0 International license.

filtered mutations with high fitness scores mapped onto a representative structure (PDB 2G15). Here mutations were filtered and mapped if the IL-3 withdrawal score "(-)IL-3" was ≥ +2 or +2.5 SD from the average wild type synonymous score, in the absence of selection the "IL-3" fitness score was ≤ 0, and that the propagated error between the (-)IL-3 and IL-3 scores was less than their difference.

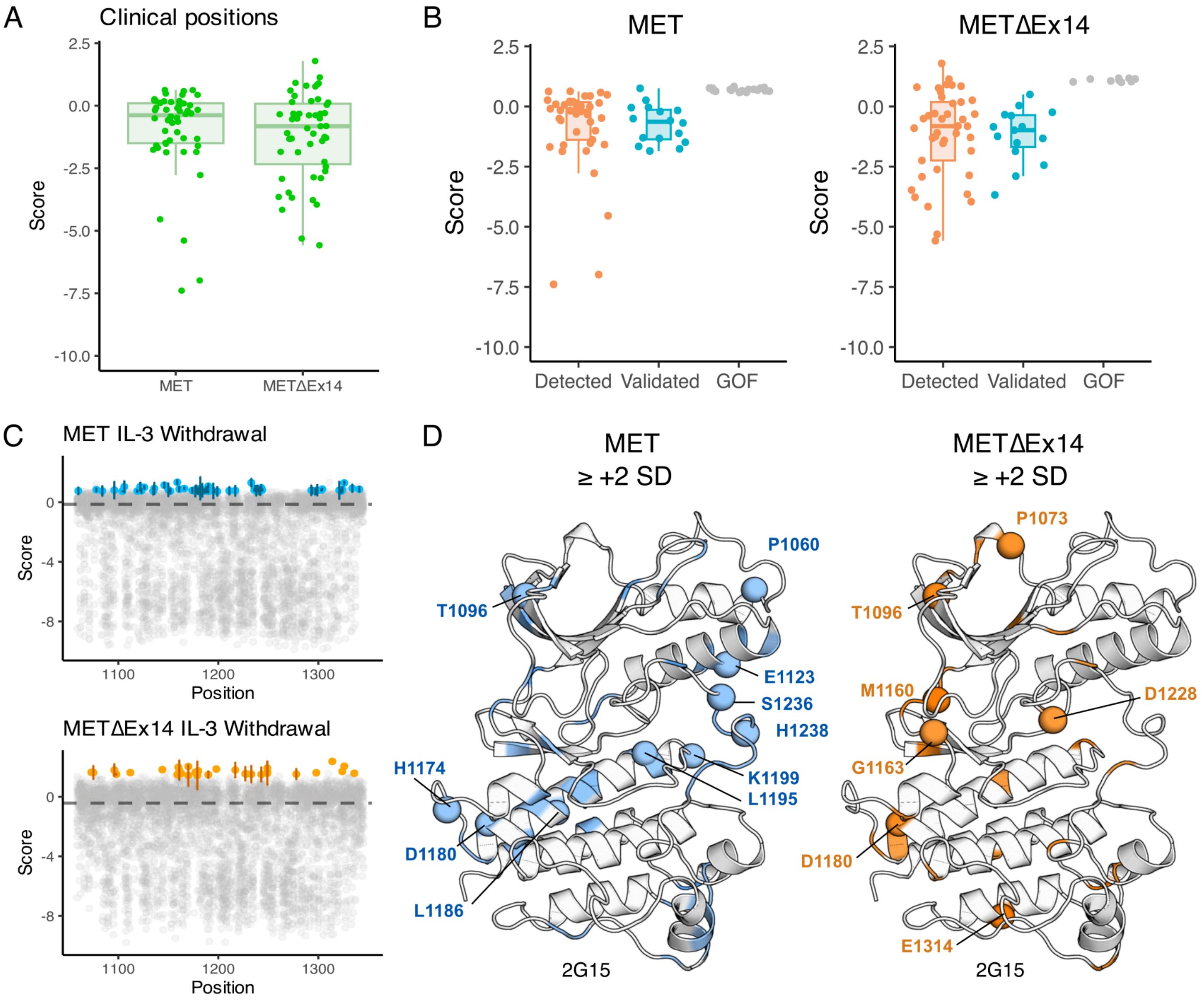

Supplemental Data Figure 8. Identification and analysis of clinically-associated mutations across MET kinase domain libraries. (A-B) Box-plots showing the distribution of mutations and fitness scores at clinically-associated residue positions annotated in cBioPortal, and further categorized based on level of literature validation (clinically detected but not validated, validated, GOF within our screen but not clinically detected). (C) Scatter plots showing the fitness scores for each mutation per kinase domain position under IL-3 withdrawal selection for TPR-MET and TPR-METΔEx14, with the average fitness scores of the wild type synonymous population of each displayed in a dashed line. Specific mutations that are ≥ +2 standard deviations (SD) from the wild type synonymous mean are highlighted with standard error displayed (TPR-MET, blue; TPR-METΔEx14, orange). (D) Structurally mapped mutations with fitness scores ≥ +2 SD form the respective wild type synonymous mean for TPR-MET and TPR-METΔEx14, with clinically-associated mutations highlighted as spheres (PDB 2G15).

bioRxiv preprint doi: https://doi.org/10.1101/2023.08.03.551866; this version posted May 7, 2024. The copyright holder for this preprint (which was not certified by peer review) is the author/funder, who has granted bioRxiv a license to display the preprint in perpetuity. It is made available under aCC-BY 4.0 International license.

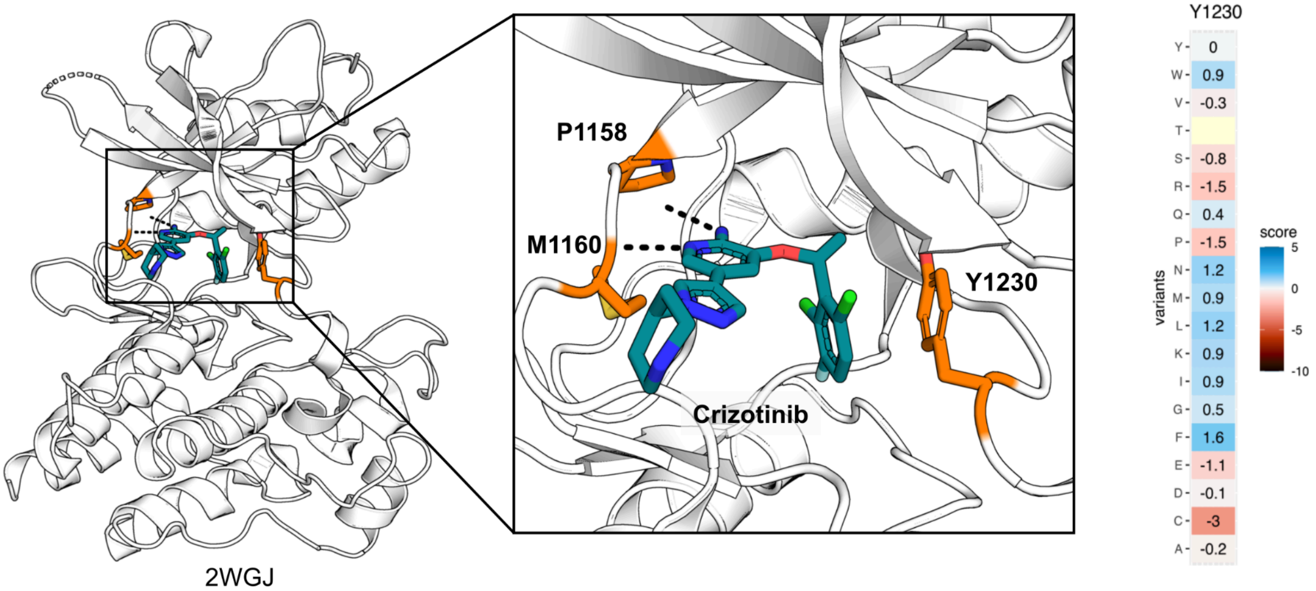

Supplemental Data Figure 9. Inhibitor-protein interactions for Y1230 and crizotinib. Crizotinib-bound MET kinase domain (2WGJ) illustrates the inhibitor-protein interactions at the active site with the loss-of-function Y1230 position annotated. Heatmap for Y1230 in METΔEx14 showing missense scores subtracted from WT synonymous.
