# Supplementary material for: Conserved regulatory motifs in the juxtamembrane domain and kinase N-lobe revealed through deep mutational scanning of the MET receptor tyrosine kinase domain
Source: bioRxiv. 2023 Aug 3:2023.08.03.551866. Preprint. [Version 1] doi: 10.1101/2023.08.03.551866 (PMC10418267; doi:10.1101/2023.08.03.551866)
Supplement: Supplementary file 1 [file media-1.pdf]

Supplemental information

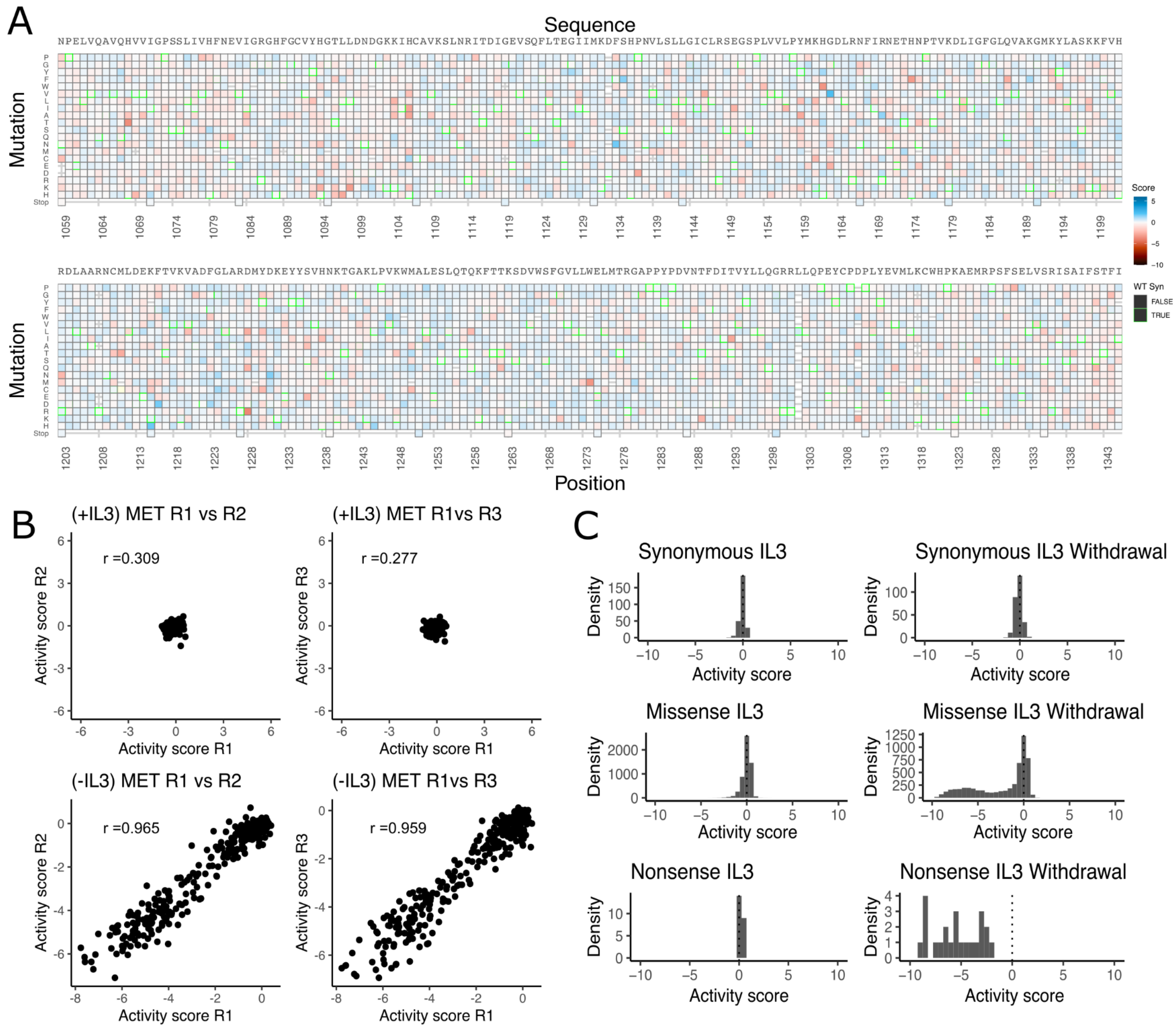

Supplemental Data Figure 1. Validation of the MET kinase domain saturation mutagensis library in IL-3 and IL-3 withdrawal selections. (A) Heatmap of MET kinase domain variants in the full-length juxtamembrane background for the IL-3 control condition that was run in parallel to the IL-3 withdrawal selection. (B) Replicate correlation analysis for both IL-3 and IL-3 withdrawal conditions, where the mean score for each position was plotted against the replicate value. The Pearson's correlation score is reported in each respective graph. (C) Distributions of synonymous, missense, and nonsense mutations for IL-3 and IL-3 withdrawal conditions.

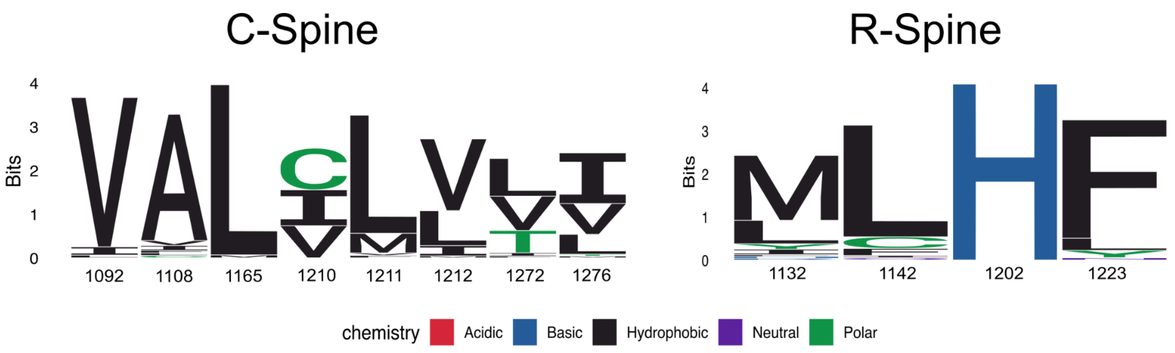

Supplemental Data Figure 2. Analysis of RTK R- and C-spine protein sequence conservation. Logo diagram representing the amino acid conservation of the C- and R-spine accross all RTK kinase domain protein sequences.

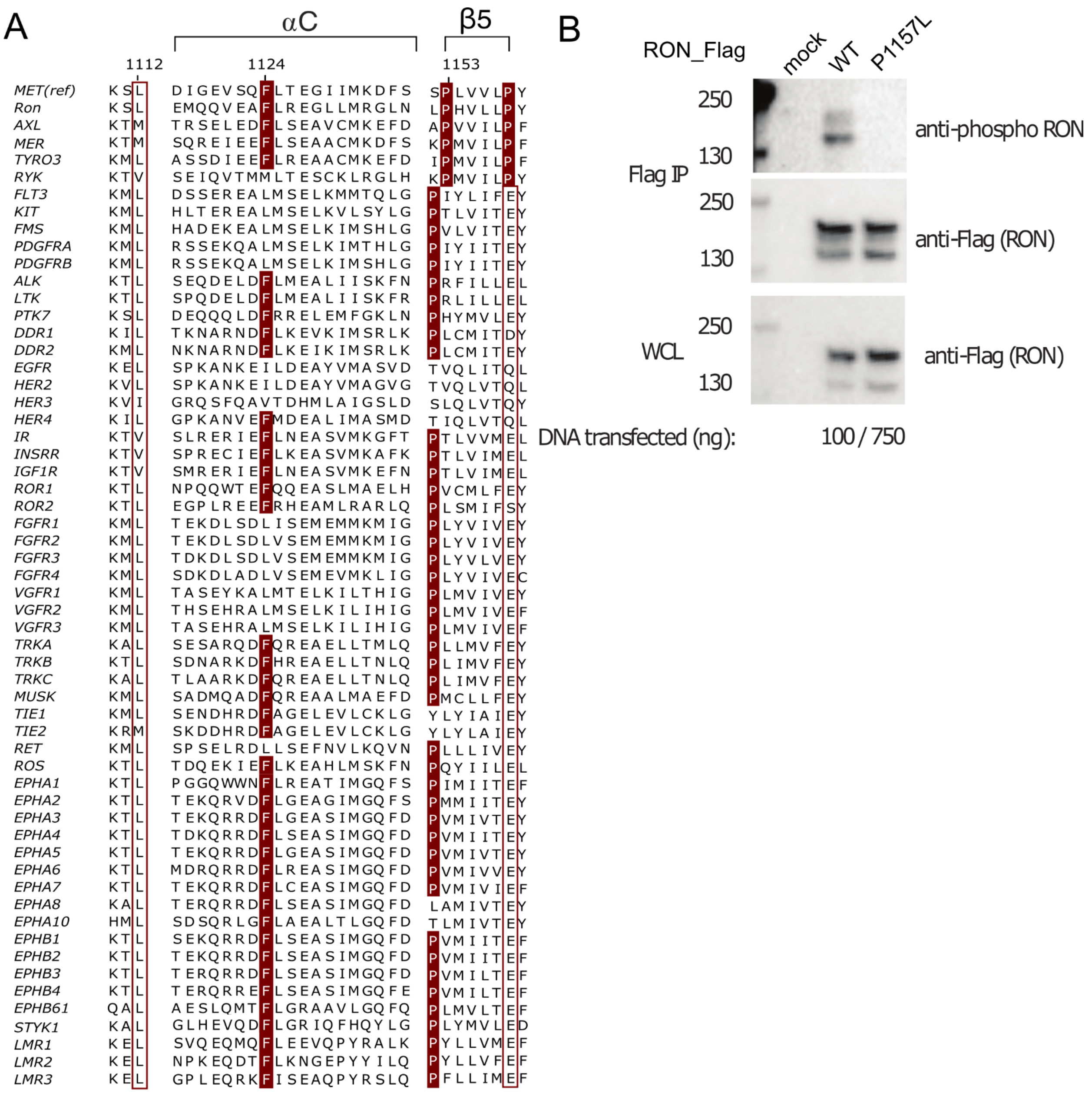

Supplemental Data Figure 3. RTK β5-turn site sequence analysis and validation in RON. (A) Protein sequence alignment of human RTK kinase domains with conservation highlighted for residues corresponding to MET positions 1112, 1124, 1153, and 1158. (B) FLAG-IP Western blot of RON with a P1157L mutation at the corresponding β5-turn site in MET expressed for 24hr in HEK293 cells with input transfection DNA concentrations for wild type RON and P1157L constructs.

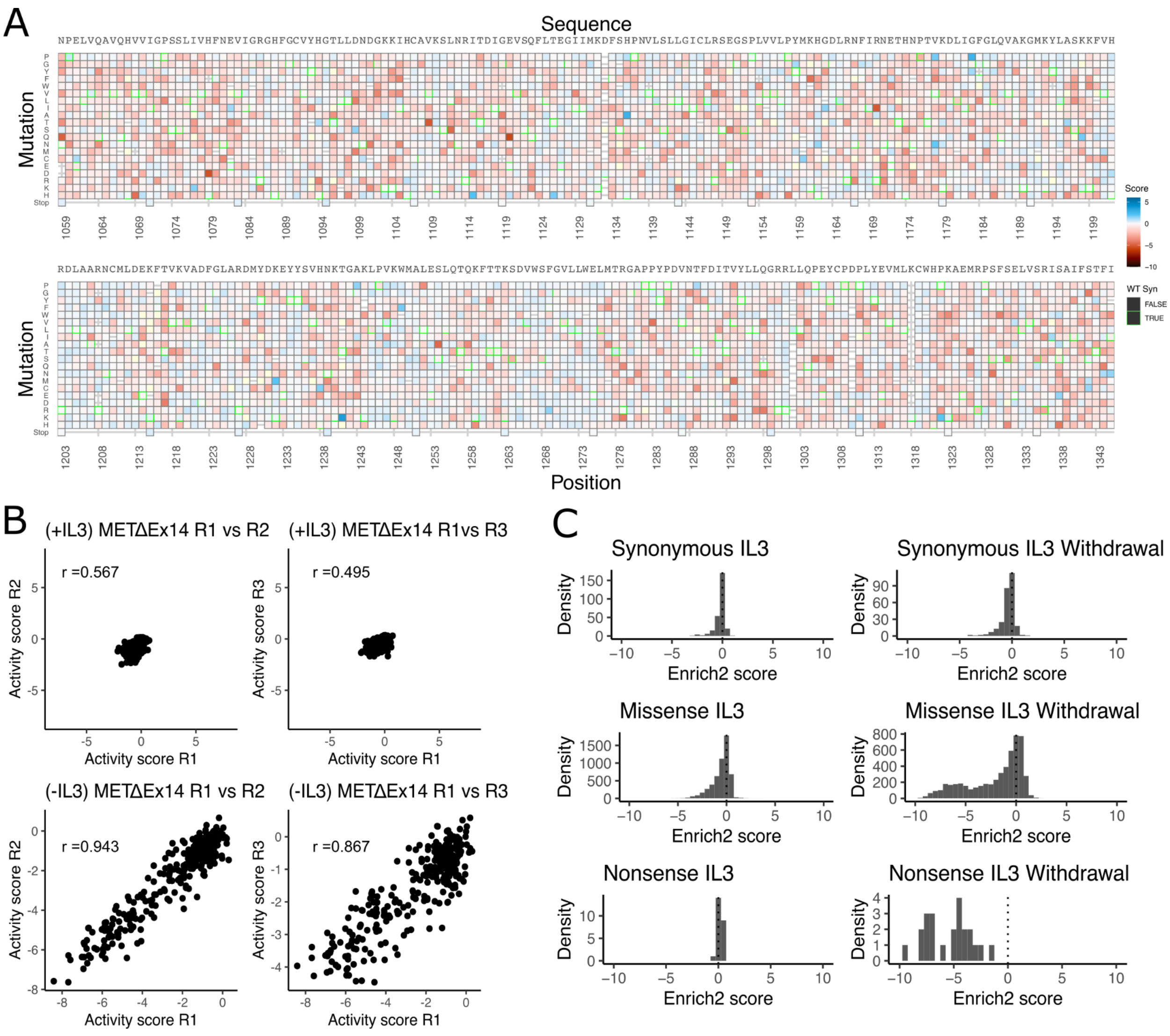

Supplemental Data Figure 4. Validation of the METΔEx14 saturation mutagensis library in IL-3 and IL-3 withdrawal selections. (A) Heatmap of METΔEx14 kinase domain variants in the IL-3 control condition that was run in parallel to the IL-3 withdrawal selection. (B) Replicate correlation analysis for both IL-3 and IL-3 withdrawal, where the mean score for each position was plotted against the replicate value. The Pearson's correlation score is reported in each respective graph. (C) Distributions of synonymous, missense, and nonsense mutations for IL-3 and IL-3 withdrawal conditions.

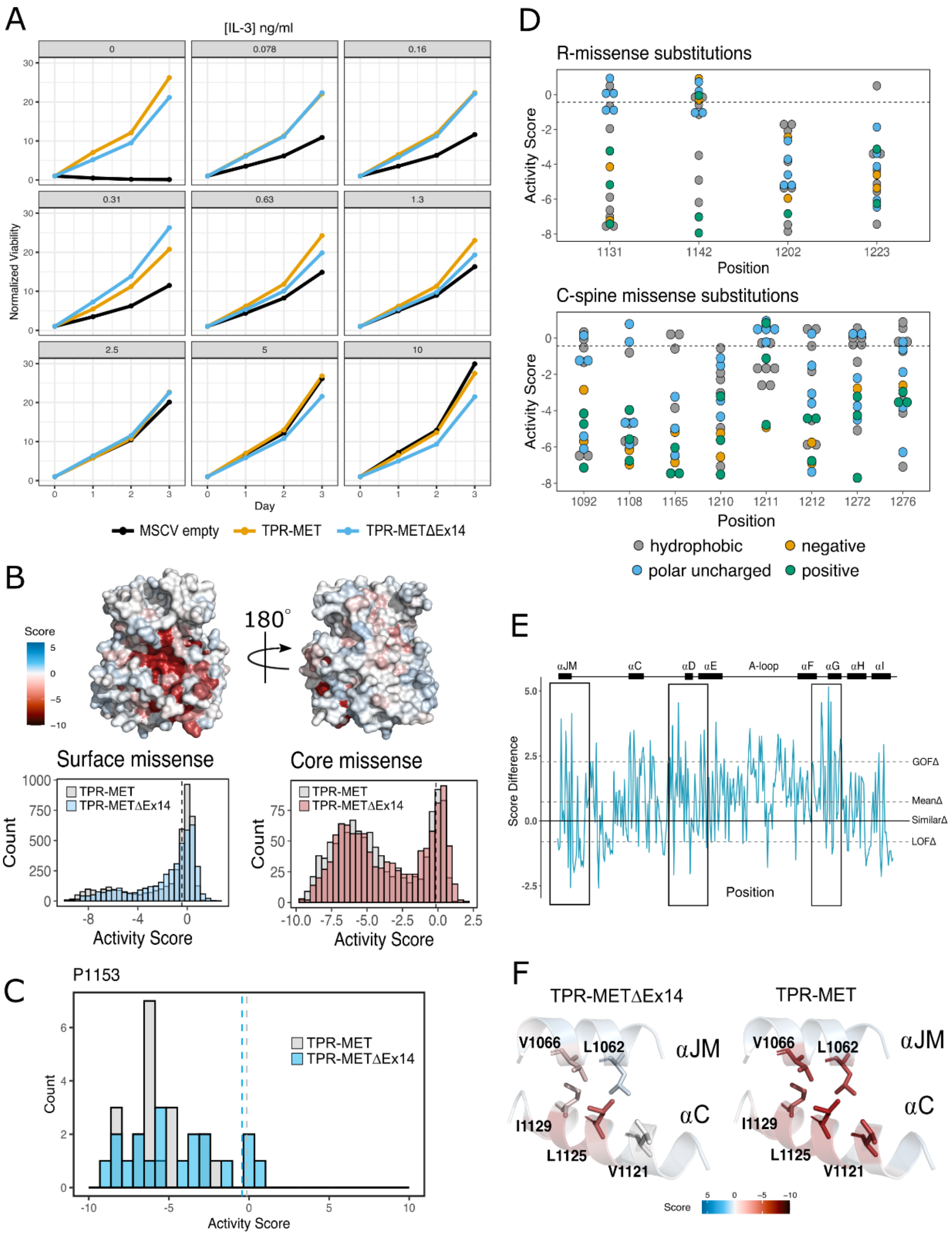

Supplemental Data Figure 5. Comparative analysis of the TPR-METΔEx14 and TPR-MET mutational landscapes. (A) IL-3 titration proliferation assay comparing Ba/F3 cells stably expressing TPR-MET, TPR-METΔEx14, and MSCV empty constructs over the course of 3 days at an IL-3 concentration range of 0, 0.078, 0.16, 0.31, 1.3, 2.5, 5, 10 ng/ml. Cell viability was normalized to day 0 for each condition. (B) Surface representation of TPR-METΔEx14 average activity scores mapped on a TPR-MET kinase domain structure (PDB 3R7O). Synonymous and nonsense mutations were left out of the averaging and surface representation. Residues at the N- and C-term that were not screened, but modeled in the crystal structure are in white and not considered in the averaging and mapping. Comparison of surface and core residues activity score distributions for TPR-METΔEx14, overlayed with the distributions for TPR-MET. A vertical dashed line in both graphs represents the mean score of WT-synonymous mutations. (C) Mutational distributions of P1153 variants for TPR-METΔEx14 (blue) and MET (gray). Dashed lines represent the mean of the WT-synonymous distribution for each library. (D) Activity scores and physiochemistry of variants shown for each residue position of the R- and C- spine of TPR-METΔEx14. (E) Mean difference plot for TPR-METΔEx14 and TPR-MET at each screened position. (E) Average score of mutations at the hydrophobic interface of αJM and αC for both TPR-METΔEx14 and TPR-MET.

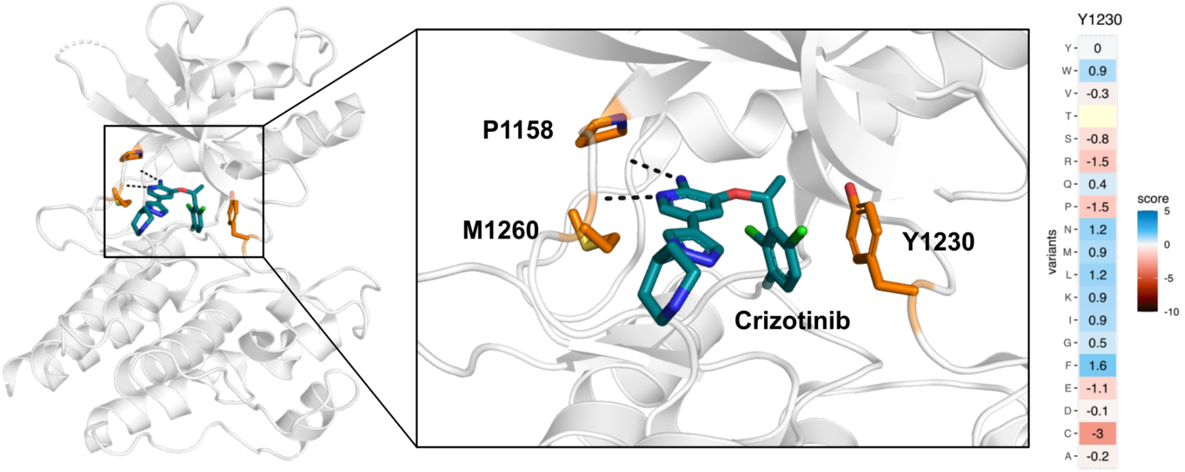

Supplemental Data Figure 6. Inhibitor-protein interactions for Y1230 and crizotinib. Crizotinib-bound MET kinase domain (2WGJ) illustrating the inhibitor-protein interactions at the active site with the loss-of-function Y1230 position annotated. Heatmap for Y1230 in METΔEx14 showing missense scores subtracted from WT synonymous.
